# Therapeutic potential of oxadiazole or furadiazole containing compounds

**DOI:** 10.1186/s13065-020-00721-2

**Published:** 2020-12-07

**Authors:** Ankit Siwach, Prabhakar Kumar Verma

**Affiliations:** Department of Pharmaceutical Sciences, Maharshi Dayanand University, Rohtak, Haryana India

**Keywords:** 1, 3, 4-oxadiazole, Heterocyclic compounds, Antiviral, Antitumor, Antitubercular

## Abstract

As we know that, Oxadiazole or furadi azole ring containing derivatives are an important class of heterocyclic compounds. A heterocyclic five-membered ring that possesses two carbons, one oxygen atom, two nitrogen atoms, and two double bonds is known as oxadiazole. They are derived from furan by the replacement of two methylene groups (= CH) with two nitrogen (-N =) atoms. The aromaticity was reduced with the replacement of these groups in the furan ring to such an extent that it shows conjugated diene character. Four different known isomers of oxadiazole were existed such as 1,2,4-oxadiazole, 1,2,3-oxadiazole, 1,2,5-oxadiazole & 1,3,4-oxadiazole. Among them, 1,3,4-oxadiazoles & 1,2,4-oxadiazoles are better known and more widely studied by the researchers due to their broad range of chemical and biological properties. 1,3,4-oxadiazoles have become important synthons in the development of new drugs. The derivatives of the oxadiazole nucleus (1,3,4-oxadiazoles) show various biological activities such as antibacterial, anti-mycobacterial, antitumor, anti-viral and antioxidant activity, etc. as reported in the literature. There are different examples of commercially available drugs which consist of 1,3,4-oxadiazole ring such as nitrofuran derivative (Furamizole) which has strong antibacterial activity, Raltegravir as an antiviral drug and Nesapidil drug is used in anti-arrhythmic therapy. This present review summarized some pharmacological activities and various kinds of synthetic routes for 2, 5-disubstituted 1,3,4-oxadiazole, and their derived products.

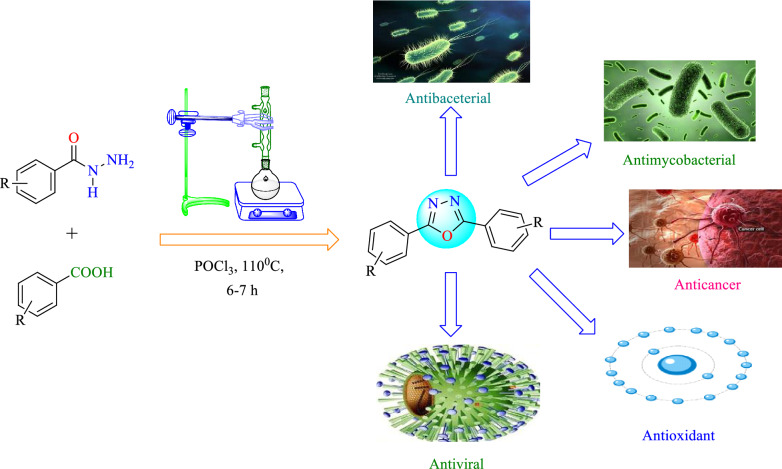

## Background

Health problems were increasing day by day and become the most serious clinical problem. Recently, medicinal chemists have been looking for new drugs to be used safely to treat these serious clinical problems. There are a lot of heterocyclic compounds that are in clinical use to treat infectious disease [1].

The most common heterocyclic are those having five or six-member fused rings and possess nitrogen, oxygen, sulfur groups as heteroatoms. Some time boron, silicon, and phosphorus atoms can be used as hetero atoms [2].

Heterocyclic compounds containing nitrogen atom such as oxadiazole moieties are of interest to researchers in the fields of medicinal and pharmaceutical chemistry [3].

A heterocycles five-member ring that possesses one oxygen, two carbons, two nitrogen atoms, and two double bonds is known as oxadiazole [4]. This type of ring system is also known as azoximes, oxybiazole, biozole, diazoxole, furadiazole, and furoxans. Oxadiazole was first synthesized in 1965 by Ainsworth through the thermolysis of hydrazine. Its molecular formula is C_2_H_2_ON_2_ and having a molecular mass of 70.05 g/mol which is soluble in water [2].

Oxadiazoles are thermally stable compounds and their calculated resonance energy is equal to 167.4 kJ/mol. The thermal stability of oxadiazoles is increased with the substitution at the second position [5].

1,3,4-oxadiazole heterocyclic ring is one of the most important heterocyclic moieties due to its versatile biological actions [6]. These are the derivatives of furan in which two methylene groups were replaced with two nitrogen atoms. Replacement of these two methylene groups by two nitrogen atoms reduces the aromaticity of the ring & the resulting oxadiazole ring exhibits conjugated diene character [7]. Another heteroatom makes a weak base to the oxadiazole due to the inductive effect [6]. Hydrogen atoms were replaced by nucleophiles which are seen in nucleophilic substitution reaction [8].

**Fig. 1:**
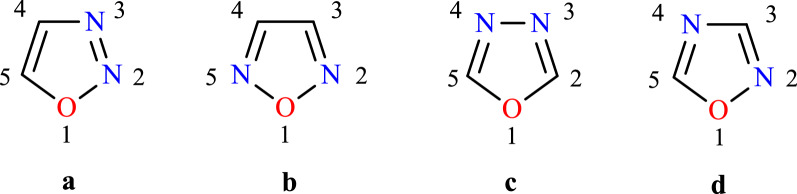
Oxadiazole

Nitrogen atoms are present in oxadiazole ring at different positions and based on the position there are four different possible isomers of oxadiazole such as 1,2,3-oxadiazole (a), 1,2,5-oxadiazole (b), 1,3,4-oxadiazole (c) and 1,2,4-Oxadiazole (d) were showed in Fig. 1 [6].

Among the different isomers, 1,3,4-oxadiazole isomer shows a lots of therapeutic activities like antibacterial [9, 10], anticonvulsant [11], antitumor [12–22], hypoglycemic, antipyretic [23], anti-tubercular [10, 24], anti-viral [25], immunosuppressive, spasmolytic, antioxidant [13, 26], anti-inflammatory [23, 27, 28], insecticidal [20], CNS stimulant, ant amoebic, antiemetic, antidepressant, anthelmintic activities, vasodilator activity, antimycotic activity [29], anti-allergic, anti-Alzheimer activity, ulcerogenic and antihypertensive activities etc. as reported in the literature [30]. Keeping the view of this, we have discussed different oxadiazole derivatives carrying urea, amide, and sulphonamide groups to investigate their anticancer, antiviral, antimicrobial, antitubercular, and antioxidant activities [31].

The presence of toxophoric –N = C–O– linkage in 1,3,4-oxadiazole ring might be responsible for their potent pharmacological activities. Among these, substituted 1,3,4-oxadiazoles are of considerable pharmaceutical interest. 2,5-disubstituted-1,3,4-oxadiazole derivatives are stable, especially 2,5-diaryl-1,3,4-oxadiazoles are more stable than the corresponding 2,5-dialkyl derivatives. Medicinal chemists have great perseverance in Research and development for the development of newer and safer antitumor agents. Tyrosine kinases (EGFR family) play a very important role in cancer proliferation. So those compounds which inhibit the activity of tyrosine kinases play a substantial role in cancer treatment. Therefore Tyrosine kinases (EGFR family) were selected and explore the binding mode of the novel compounds to EGFR tyrosine kinase active site [32].

There is various kind of synthetic route from which we can synthesize 1,3,4-oxadiazole, and their derived products. In general, 1,3,4-oxadiazole can be synthesized by the reaction of acid hydrazide or hydrazine along with carboxylic acids/acid chlorides and direct ring closure of diacyl hydrazines employing different kinds of the cyclizing agent such as phosphorus oxychloride, thionyl chloride, phosphorus pentaoxide, triflic anhydride, polyphosphoric acid, acetic anhydride and the direct reaction of an acid with (N-isocyananimino-) triphenylphosphorane [33]. In some reaction, carbon disulfide is also used for ring closure [34].

**Fig. 2:**
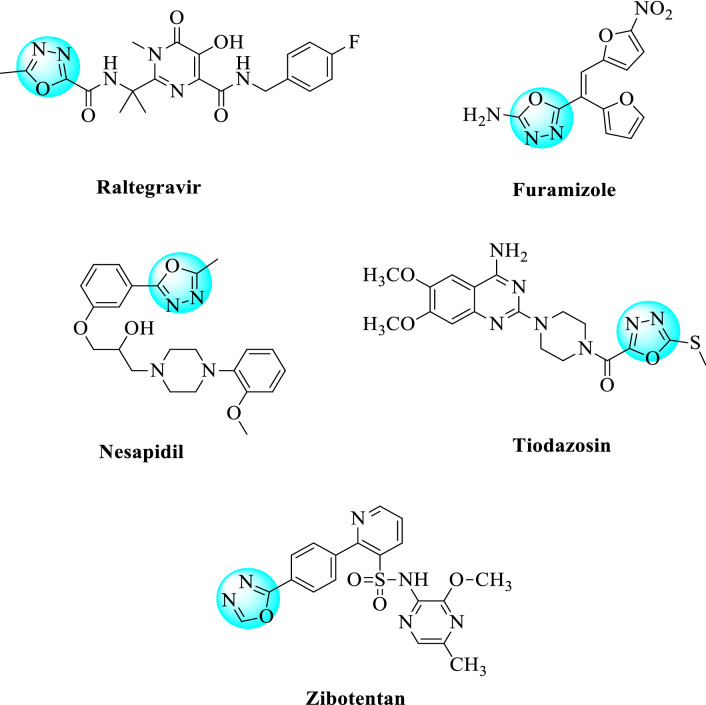
Commercially available drugs which contain 1,3,4-oxadiazole nucleus

There are different examples of commercially available drugs containing 1,3,4-oxadiazole ring (Fig. 2) such as a nitrofuran derivative (Furamizole) which has strong antibacterial activity [35]. Raltegravir as an antiviral drug and Nesapidil drug is used in anti-arrhythmic therapy. The FDA approved anticancer agent Zibotentan is a 1,3,4-oxadiazole nucleus containing the most privileged derivatives available in the market [36]. Tiodazosin is used as an antihypertensive agent [37]. This present review summarized some pharmacological activities and various kinds of synthetic routes for 2,5-disubstituted 1,3,4-Oxadiazole, and their derived products during the last decade (2005–2020).

### The mechanism for the formation of 2,5-disubstituted 1,3,4-oxadiazole

**Fig. 3:**
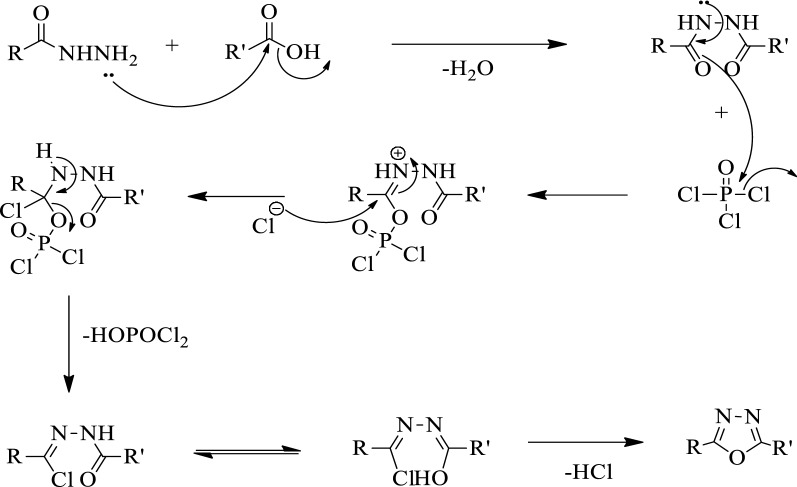
Mechanism for the formation of 2,5-disubstituted 1,3,4-oxadiazole using phosphorus oxychloride

The probable mechanism for the formation of the 1,3,4-oxadiazole is given in (Fig. 3). The presence of lone pair of electron on the nitrogen atom of acid hydrazide attacks the carbonyl carbon atom of carboxylic acid eliminates a water molecule to form a hydrazide derivative which further reacts with phosphorus oxychloride, undergoes ring closure with the elimination of hydrogen chloride, and form 1,3,4-oxadiazole ring [38].

### Structure–activity relationship of 1,3,4-oxadiazole derivatives

**Fig. 4:**
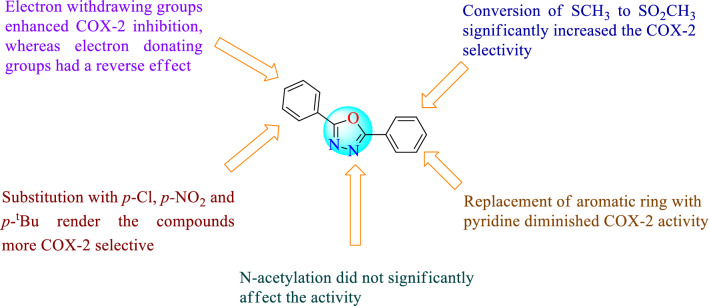
Structure–activity relationship of 1,3,4-oxadiazole

The structure–activity relationship of 1,3,4-oxadiazole is given in (Fig. 4). Substitution of phenyl ring with different substituents like *p*-Cl, *p*-NO_2_ & *p*-^t^Bu further increases the activity. The conversion of the methylthio group into the methyl-sulfonyl group also increases the activity. The replacement of the phenyl ring along with the pyridine ring decreases the activity. If the acetyl group is present on the nitrogen atom of the oxadiazole ring did not significantly affect the activity [39]. Thus, based on the aforementioned results, we hypothesized that 2,5-disubstituted 1,3,4-oxadiazole scaffold may lead to novel potent agents with broad biological activity profile and improved pharmacokinetic properties.

### Pharmacological profile of some oxadiazole derivatives

**Fig. 5:**
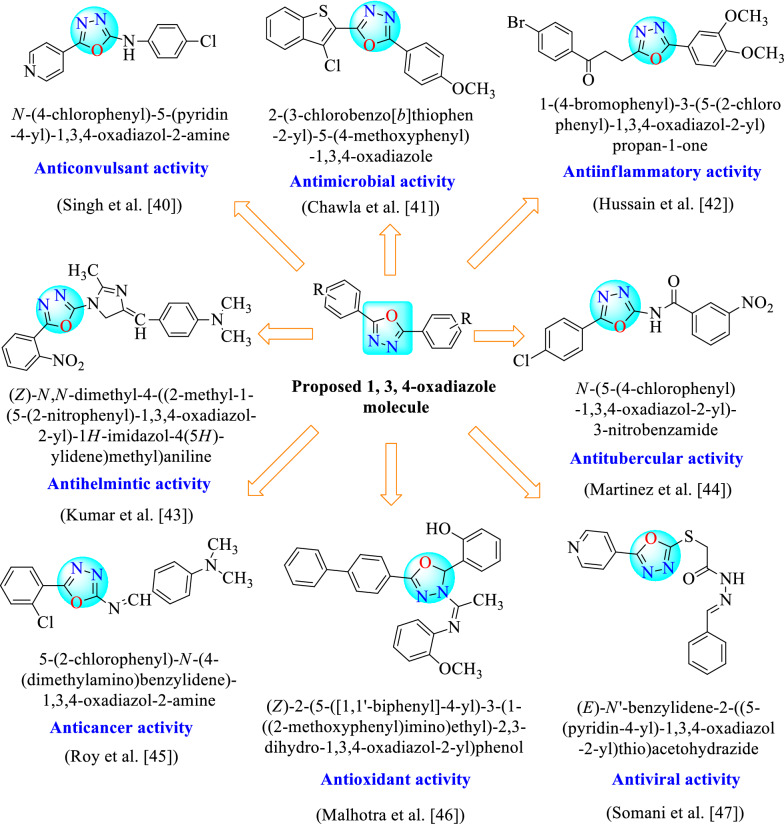
Therapeutic activity of 1,3,4-oxadiazole nucleus

Compound N-(4 chlorophenyl) amino-5-(4-pyridyl)- 1,3,4-oxadiazole having electron-withdrawing group shows better anticonvulsant activity [40]. Compounds with p-methoxy group increase the antimicrobial potential [41] and 3, 4-dimethoxy containing compound increase anti-inflammatory activity as compared to reference drug [42]. 1,3,4-Oxadiazole nucleus containing compounds along with different substituents shows various kinds of activities (Fig. 5).

## Antimicrobial activity

**Scheme 1:**
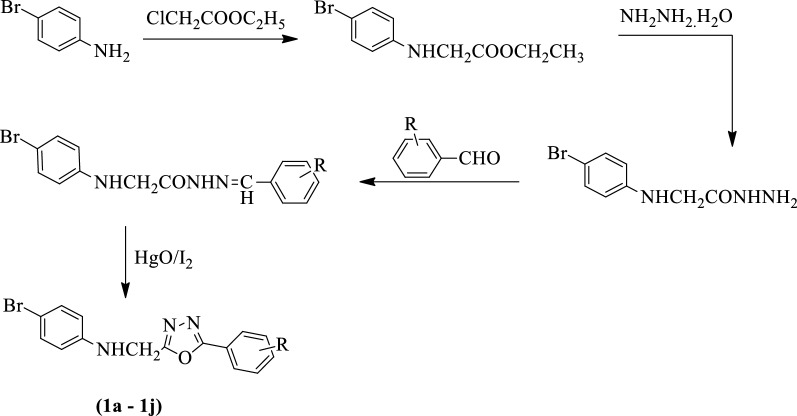
Synthesis of substituted 1,3,4-oxadiazole (1a-j) with 4-bromoaniline starting material

Bhat et al. [48] developed *4-bromo-N-[(5-(substituted phenyl)-1,3,4-oxadiazol-2yl)methyl]aniline* (Scheme 1) and these derivatives were screened for antimicrobial activity against *S. aureus, E. coli*, *B. Subtilis,* and *P. aeruginosa* using amoxicillin as a positive control. The antimycotic activity was evaluated for these compounds against *A. niger* and *C. albicans* using ketoconazole as a reference standard. Derivatives with different groups like -OH, -NO_2_ **[1b, 1c, 1d, 1g]** shows good antimicrobial activity against fungal strains. Derivatives with groups like p-methoxy, p-chloro, p-methyl **[1e, 1f, 1h]** show better antimicrobial potential as compared to amoxicillin. The results of the antimicrobial activity of synthesized 1,3,4-oxadiazole derivatives were presented in (Table 1, Bhat et al. [48]).

**Table 1:** Antimicrobial activity of titled compounds (1a-j) [48]

Compound	Diameter of zone of inhibition (mm)				
	*S. aureus*	*B. subtilis*	*E. coli*	*P. aeruginosa*	*C. albicans*
1a 1b 1c 1d 1e 1f 1 g 1 h 1i 1j Amoxicillin Ketoconazole	13 14 14 15 18 19 14 18 16 15 21 –	15 14 15 14 19 17 12 18 15 14 22 –	14 13 14 13 18 18 15 19 14 15 21 –	13 12 15 13 15 16 10 15 13 12 22 –	08 15 14 15 08 09 15 09 10 11 – 23

**Scheme 2:**
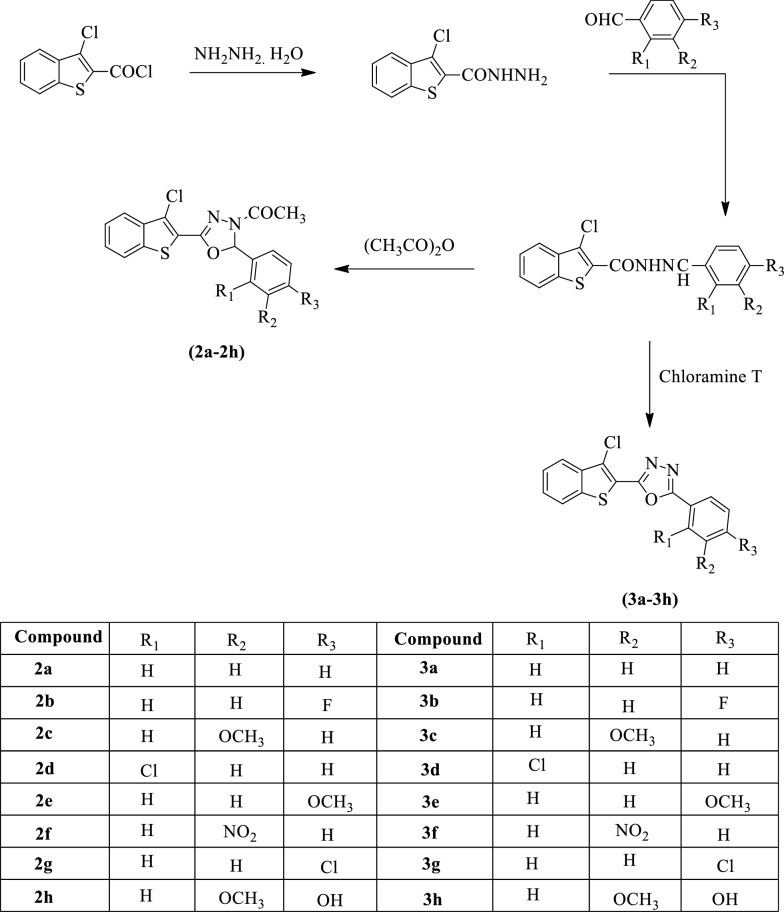
Synthesis of substituted 1,3,4-oxadiazole derivatives

Chawla et al. [41] developed *1-(5-(3-chlorobenzo[b]thiophen-2-yl)-2-(2,3,4-trisubstituted phenyl)-1,3,4-oxadiazol-3(2H)-yl)ethanone* and *2-(3-chlorobenzo[b]thiophen-2-yl)-5-(2,3,4-trisubstituted phenyl)-1,3,4-oxadiazole* by using Scheme 2. The antibacterial activity of synthesized derivatives was evaluated against different bacterial strains such as (*S. aureus, B. Subtilis, E. coli*, and *P. aeruginosa*) using ciprofloxacin as standard drug. The antimycotic activity of these derivatives was evaluated against *A. niger* and *C. albicans* using fluconazole as a reference standard and the results were summarized in (Table 2, Chawla et al. [41]).

**Table 2:** Antimicrobial activity of titled compounds (2a-h) and (3a-h) [41]

Compound	Diameter of zone of inhibition (mm)					
	Antibacterial activity				Antifungal activity	
	*S. aureus*	*B. subtilis*	*E. coli*	*P. aeruginosa*	*C. albicans*	*A. niger*
2a	14	21	10	17	09	10
2b	18	19	12	15	10	11
2c	30	27	14	18	09	11
2d	19	22	11	18	10	11
2e	28	28	14	14	10	09
2f	14	19	10	15	10	10
2g	21	23	13	19	11	09
2h	14	20	10	16	09	10
3a	11	12	10	09	11	11
3b	10	12	09	11	12	12
3c	20	21	12	13	11	11
3d	20	22	16	18	10	11
3e	18	19	11	13	11	10
3f	11	13	10	11	10	11
3g	12	14	09	12	10	10
3h	10	13	09	11	10	11
Ciprofloxacin	26	26	28	25	–	–
Fluconazole	–	–	–	–	26	25

**Scheme 3:**
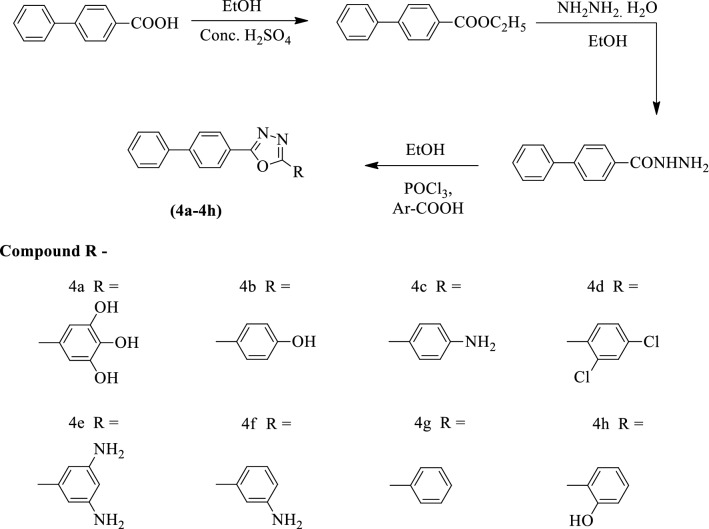
Synthesis of substituted 1,3,4-oxadiazole with 4-biphenyl carboxylic acid as starting material

Kumar et al. [43] developed *2-((1, 1′-biphenyl)-4-yl)-5-(substituted phenyl)-1,3,4-oxadiazole* by using Scheme 3. The antibacterial activity of these derivatives was evaluated against different Gram + ve (*S. aureus*) and Gram -ve (*K. pneumonia, E. coli*, and *P. aeruginosa*) strains using ofloxacin as a reference standard. The cup plate agar diffusion method was used for the determination of the zone of inhibition. The results of antibacterial activity were summarized in (Table 3, Kumar et al. [43]).

**Table 3:** In vitro antimicrobial activity of the titled compounds (4a-4 h) [43]

Compound	Diameter of zone of inhibition (mm)			
	Antibacterial activity			
	*S. aureus*	*P. aeruginosa*	*K. pneumonia*	*E. coli*
4a	19	17	18	19
4b	17	16	17	15
4c	14	13	16	17
4d	21	19	19	20
4e	12	11	13	12
4f	13	14	15	12
4g	12	13	11	11
4h	17	16	15	17
Ofloxacin	41	38	39	37

**Scheme 4:**
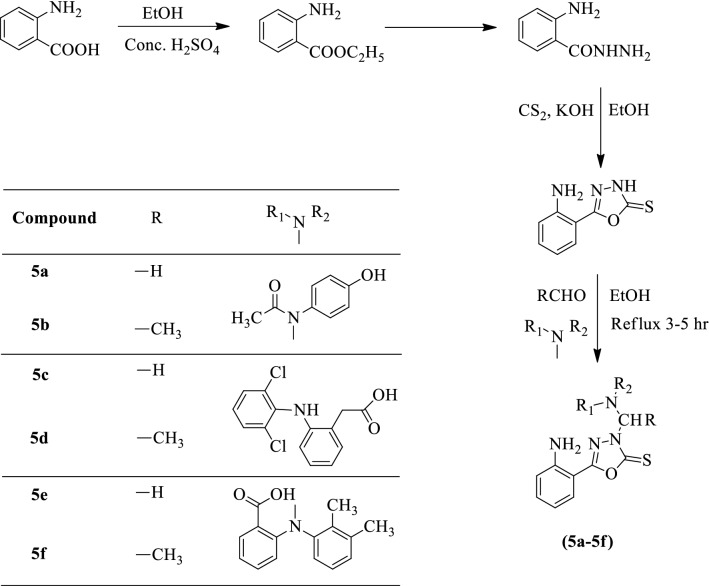
Synthesis of substituted 1,3,4-oxadiazole with 2-aminobenzoic acid as starting material

Kanthiah et al. [5] developed *5-(2-aminophenyl)-3-(substituted (disubstituted amino) methyl)-1,3,4-oxadiazole-2(3H)-thione* by using Scheme 4. The antimicrobial activity of synthesized derivatives was evaluated against different two Gram + ve (*S. aureus* and *S. pyogenes*) and Gram -ve (*E. coli* and *K. aerogenes*) strains using amikacin as a reference standard. The antimycotic activity was also evaluated for these derivatives against *C. albicans* using ketoconazole as positive control and the results were summarized in (Table 4, Kanthiah et al. [5]).

**Table 4:** Antimicrobial activity of the titled compounds (5a-5f) [5]

Compound	Diameter of zone of inhibition (mm)				
	Antibacterial activity				Antifungal activity
	*S. aureus*	*S. pyrogenes*	*E. coli*	*P. aeruginosa*	*C. albicans*
5a	10	13	12	08	14
5b	13	11	14	09	12
5c	12	13	15	09	14
5d	12	11	13	10	13
5e	09	09	10	07	11
5f	08	09	09	06	10
Amikacin	16	15	17	18	–
Ketoconazole	–	–	–	–	18

**Scheme 5:**
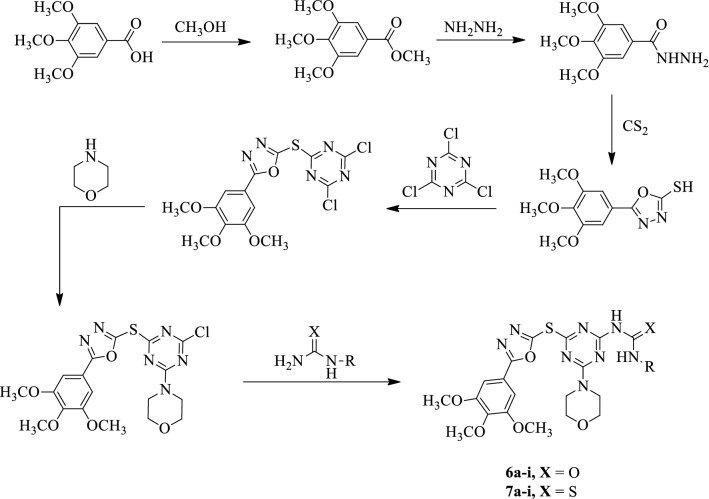
Synthesis of substituted 1,3,4-oxadiazole with 3, 4, 5-trimethoxybenzoic acid as starting material

Chikhalia et al. [49] developed *1-substituted-3-(4-morpholino-6-((5-(3,4,5-trimethoxyphenyl)-1,3,4-oxadiazol-2-yl)thio)-1,3,5-triazin-2-yl)substituted urea* (Scheme 5) and evaluated for antimicrobial activity against different strains such as (*Staphylococcus aureus*, *Bacillus subtilis*, *Escherichia coli*, and *Pseudomonas aeruginosa*) using ampicillin as a reference standard. The antifungal activity was also evaluated for these derivatives against *C. albicans* using fluconazole as a reference standard. Compound **6e** shows better activity against *E. coli* and *P. aeruginosa* as compared to a positive control (ampicillin). Compound **6 g** also shows better activity towards *P. aeruginosa* but lower than that of ampicillin. Compound **7c** and **7g** showed good activity against *C. albicans* but slightly lower than that of fluconazole. The results of antimicrobial activity were shown in (Table 5, Chikhalia et al. [49]).

**Table 5:** Minimum inhibitory concentration (MIC) of titled compounds [49]

Compound			*S. aureus*	*B. subtilis*	*P. aeruginosa*	*E. coli*	*C. albicans*
	R	X	ATCC 25923	ATCC 6633	ATCC 27853	ATCC 27853	ATCC 10231
6a	C6H5	O	0.3	0.15	0.15	1.25	2.5
6b	2-CH_3_ C_6_H_5_	O	0.31	0.07	1.25	0.625	5.0
6c	3-CH_3_ C_6_H_5_	O	0.625	0.15	5.0	2.5	10
6d	4-CH_3_ C_6_H_5_	O	2.5	2.5	0.03	5.0	1.25
6e	2-Cl C_6_H_5_	O	0.15	1.25	0.019	0.019	5.0
6f	3-Cl C_6_H_5_	O	0.15	0.625	1.25	1.25	2.5
6g	4-Cl C_6_H_5_	O	0.15	0.3	0.019	0.07	0.15
6h	3-NO_2_ C_6_H_5_	O	-	10	1.25	–	–
6i	4-NO_2_ C_6_H_5_	O	2.5	–	0.625	5.0	10
7a	2-CH_3_ C_6_H_5_	S	1.25	–	2.5	10	–
7b	4-CH_3_ C_6_H_5_	S	1.25	5.0	2.5	1.25	5.0
7c	3-OH C_6_H_5_	S	2.5	1.25	0.019	2.5	10
7d	4-OH C_6_H_5_	S	0.15	0.625	2.5	0.625	1.25
7e	4-Cl C_6_H_5_	S	0.625	0.07	5.0	0.03	0.31
7f	3-NO_2_ C_6_H_5_	S	2.5	2.5	10	1.25	2.5
7g	4-NO_2_ C_6_H_5_	S	2.5	5.0	5.0	0.1	0.15
Ampicillin			0.019	0.005	0.005	0.01	–
Fluconazole			–	–	–	–	0.01

## Antitumor activity

**Scheme 6:**
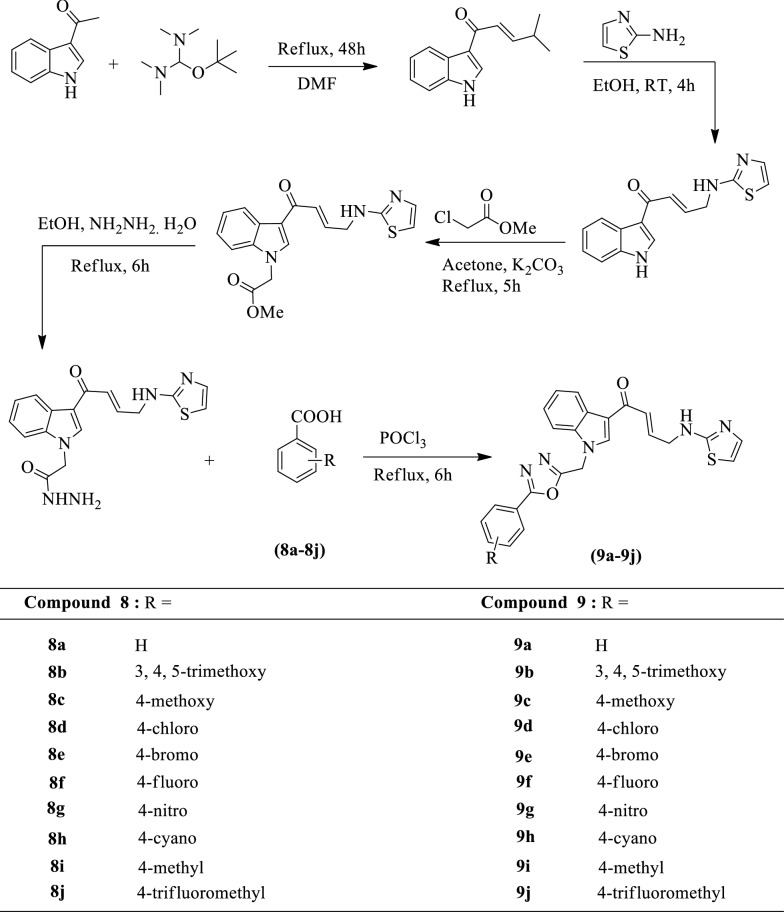
Synthesis of substituted 1,3,4-oxadiazole derivatives

Srinivas et al. [30] developed *(E)-1-(1-((5-substituted-1,3,4-oxadiazol-2-yl)methyl)-1H-indol-3-yl)-4-(thiazol-2-ylamino)but-2-en-1-one* (Scheme 6) and evaluated for antitumor activity by MTT assay against four different cancer cell lines such as HT-29 (colon), A375 (melanoma), MCF-7 (breast) and A549 (lung) using combretastatin-A4 as reference standard. All derivatives of 1,3,4-oxadiazole fused indole ring was showed a variable degree of anticancer activity along with IC_50_ values ranging from 0.010 ± 0.004 and 18.50 ± 0.86 μM. Among the different derivatives **9a, 9b, 9f, 9g, 9h**, and **9j** were exhibited more potent than the positive control. The results of antitumor activity were presented in (Table 6, Srinivas et al. [30]).

**Table 6:** In vitro cytotoxicity (IC_50_Μ)^a^ data of compounds (9a-j) [30]

Compound	A549^bc^	MCF-7^d^	A375^e^	HT-29^f^
9a	1.20 ± 0.16	0.098 ± 0.004	2.56 ± 0.36	0.012 ± 0.001
9b	0.023 ± 0.006	0.011 ± 0.001	–	1.90 ± 0.71
9c	2.30 ± 0.21	2.19 ± 0.28	–	8.30 ± 1.60
9d	3.56 ± 0.19	2.11 ± 0.23	6.13 ± 1.12	7.14 ± 0.86
9e	5.02 ± 1.02	12.4 ± 0.96	–	–
9f	0.27 ± 0.02	1.07 ± 0.59	2.81 ± 0.25	1.55 ± 0.65
9 g	0.013 ± 0.001	0.80 ± 0.15	1.05 ± 0.53	1.24 ± 0.17
9 h	1.02 ± 0.50	0.010 ± 0.004	1.99 ± 0.29	3.78 ± 0.16
9i	13.9 ± 0.54	18.50 ± 0.86	8.23 ± 1.35	–
9j	0.90 ± 0.09	0.12 ± 0.01	0.39 ± 0.012	1.10 ± 0.54
Combretastatin-A4	0.11 ± 0.01	0.18 ± 0.01	0.21 ± 0.02	0.93 ± 0.03

^a^Each data represented as mean ± S.D values. From three different experiments performed in triplicates, ^bc^A549: Human lung cancer cell line, ^d^MCF-7: Human breast cancer cell line, ^e^A375: Human melanoma cancer cell line, ^f^HT-29: Human colon cancer cell line. –: Not active

**Scheme 7:**
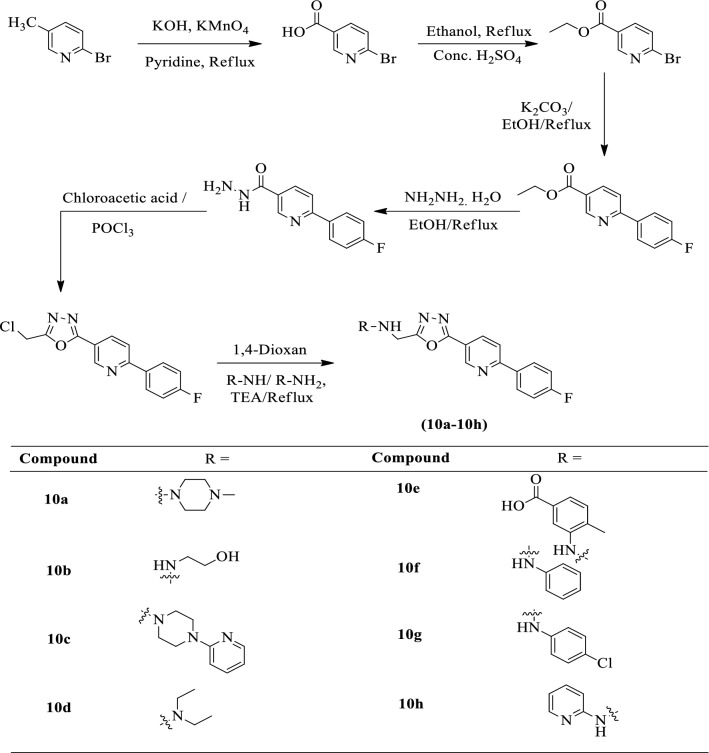
Synthesis of substituted 1,3,4-oxadiazole derivatives

Vinayak et al. [50] developed *N-[(5-(6-(4-fluorophenyl)pyridine-3-yl)1,3,4-oxadiazol-2-yl)methyl]-substituted-1-amine* by using Scheme 7 and evaluated for antiproliferative activity against different cell lines such as HeLa, HepG2, and Caco by MTT assay using 5-Fluorouracil as a reference standard. The derivative **10a** and **10d** showed excellent activity against HepG2 cell lines. The compound **10f** gives better results against the Caco-2 cancer cell line. The results of the anti-proliferative activity of synthesized derivatives were showed in (Table 7a, b, and c, Vinayak et al. [50]).

**Table 7:** (a) IC_50_ values of the synthesized novel amine derivatives. (b) CC_50_ values of the synthesized novel amine derivatives. (c) Selectivity index (SI) of the synthesized novel amine derivatives [50]

Panel (a)				
Compound	IC_50_^#^values of 10(a-h) in (μM)			
	HeLa	Caco-2	HepG2	
10a	212.4 ± 1.2	203.6 ± 2.3		2.6 ± 0.5
10b	85.6 ± 0.8	112.5 ± 1.2		45.6 ± 1.1
10c	34.8 ± 1.3	123.8 ± 1.4		128.9 ± 3.5
10d	112.9 ± 0.4	145.6 ± 0.4		5.8 ± 1.6
10e	118.4 ± 0.5	212.3 ± 0.4		32.2 ± 0.3
10f	78.3 ± 5.4	2.3 ± 0.5		23.5 ± 4.6
10 g	56.4 ± 3.4	56.8 ± 1.2		156.7 ± 2.3
10 h	88.6 ± 1.2	34.6 ± 0.9		176.4 ± 1.6
5-FU	7.6 ± 0.3	8.8 ± 0.6		7.6 ± 0.2

Panel (b)				
Compound	CC_50_^*^ of the compound 10(a-h) in (μM)			
	HeLa	Caco-2	HepG2	
10a	120 ± 1.2	112 ± 1.3		34 ± 0.5
10b	7.6 ± 0.6	145 ± 1.1		129 ± 0.3
10c	200	178 ± 2.3		102 ± 1.1
10d	450	100 ± 2.6		112 ± 1.4
10e	56 ± 2.4	62 ± 1.2		76 ± 3.4
10f	127 ± 3.4	87 ± 2.6		77 ± 0.4
10 g	200	23 ± 1.5		91 ± 4.3
10 h	123 ± 2.3	156 ± 0.4		73 ± 1.4
5-FU	57 ± 0.3	69 ± 2.3		52 ± 1.8

Panel (c)				
Compound	SI of the compound 10(a-h)			
	HeLa	Caco-2	HepG2	
10a	0.566	0.551		13.06
10b	0.887	1.288		2.828
10c	5.747	1.437		0.791
10d	3.985	0.686		19.31
10e	0.472	0.292		0.236
10f	1.621	37.8		3.276
10g	3.546	0.404		0.580
10h	1.388	4.508		0.413
5-FU	7.5	7.84		6.84

*Concentration of compound at 50% of the remaining viable cells

^#^Inhibitory concentration at 50% of the viable cells

± Average value of the two independent experiments

**Scheme 8:**
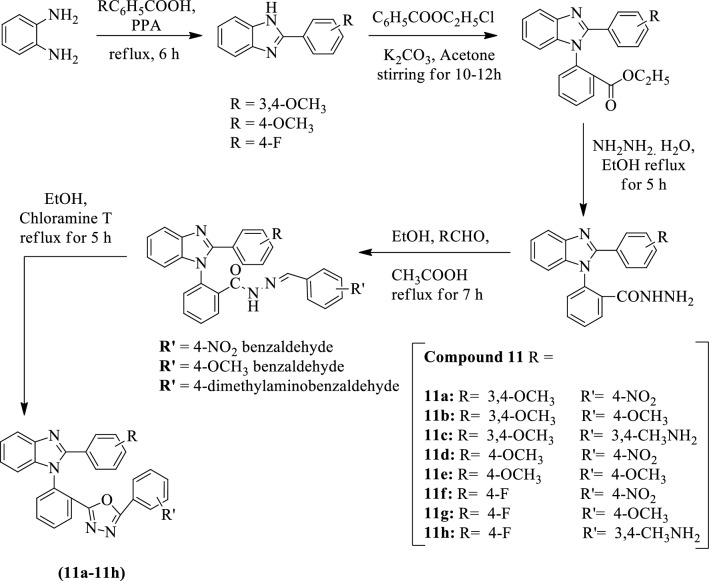
Synthesis of substituted 1,3,4-oxadiazole with benzene 1, 2-diamine as starting material

Kapoor et al. [51] developed *2-(substituted phenyl)-5-(2-(2-(substituted phenyl)-1H-benzo[d]imidazol-1-yl)phenyl)-1,3,4-oxadiazole* by using Scheme 8 and evaluated for antitumor activity against MCF-7 (breast) cancer cell line by MTT assay. Compound **11e** shows better cytotoxic activity as compare to **11a**, **11b,** and **11c**. Compounds **11f, 11g, 11h** also show the excellent cytotoxic activity as compared to the rest of the derivatives. Compounds **11e** and **11h** flourished potent cytotoxic activity with minimum percentage viability. Each compound was tested to calculate the percentage viability of cell line against the different concentrations which is presented in (Table 8, Kapoor et al. [51]).

**Table 8:** In-vitro cytotoxicity of synthesized compounds against Breast cancer cell line (MCF-7) [51]

Compound	% Viability				
	6.25 μg/ml	12.5 μg/ml	25 μg/ml	50 μg/ml	100 μg/ml
11a	38.04	37.15	39.68	35.11	40.31
11b	38.26	42.70	37.90	38.84	43.24
11c	44.35	41.6	41.81	39.64	37.24
11d	42.70	39.46	40.48	37.61	37.37
11e	30.60	32.20	34.48	33.86	37.54
11f	32.57	33.09	30.88	30.75	24.87
11 g	34.39	33.58	28.80	32.40	30.96
11 h	32.03	35.40	31.25	33.69	34.45

Control % viability = 100

**Scheme 9:**
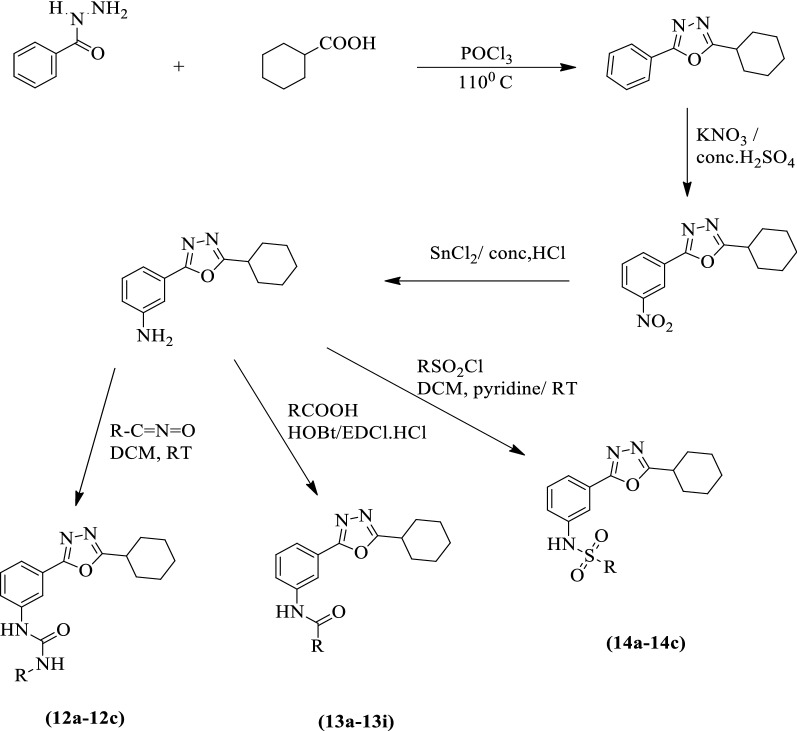
Synthesis of 1,3,4-oxadiazole derivatives

Kavitha et al. [31] developed *N-substituted-(3-(5-cyclohexyl-1,3,4-oxadiazol-2-yl)phenyl)benzamide, urea*, and *substituted benzenesulfonamide* derivatives by using Scheme 9. The anticancer activity of synthesized derivatives was evaluated against different cancer cell lines like HeLa and MCF-7 using cisplatin as a reference standard. Among the different derivatives, compounds **12a, 12b, 12c, 13c, 13d**, and **14b** showed significant activity after 48 h exposures. Further derivatives **12a, 13c, 13d**, and **14b** also showed excellent antitumor activity as compared to the positive control. Compound **12b** showed excellent antitumor activity as compared to the rest of other compounds. The results of the antitumor activity of these derivatives were presented in (Table 9, Kavitha et al. [31]).

**Table 9:** Preliminary cytotoxicity screening of synthesized 1,3,4-oxadiazole derivatives [31]

Compound	IC_50_ μM	
	HeLa	MCF-7
12a	79.7	81.6
12b	30.4	23.5
12c	45.6	28.6
13a	≥ 100	≥ 100
13b	≥ 100	≥ 100
13c	80.1	78.3
13d	58.8	62.4
13e	≥ 100	≥ 100
13f	100.3	≥ 100
13 g	≥ 100	≥ 100
13 h	≥ 100	≥ 100
13i	≥ 100	≥ 100
14a	≥ 100	≥ 100
14b	62.9	60.9
14c	≥ 100	≥ 100
Standard	3.5	3.5

**Scheme 10:**
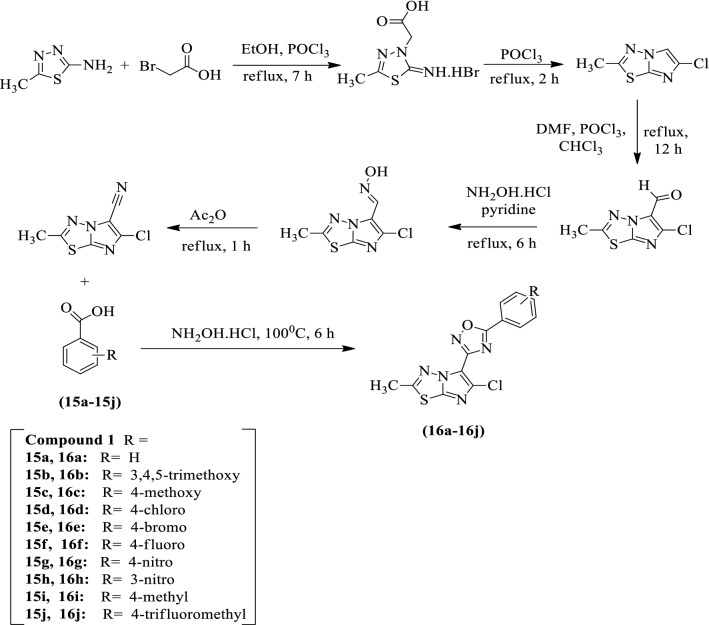
Synthesis of 1,2,4-oxadiazole derivatives

Chakrapani et al. [52] developed *3-(6-chloro-2-methylimidazo[2,1-b][1,3,4]thiadiazol-5-yl)-5-(substituted phenyl)-1,2,4-oxadiazole* by using Scheme 10. The antitumor activity of the synthesized derivatives was evaluated by MTT assay against ACHN (renal), MCF-7 (breast), and A375 (melanoma) tumor cell line using doxorubicin as a reference standard. The compound **16b** shows good cytotoxic activity in comparison to the reference drug. The compound **16j** exhibits excellent activity towards melanoma cancer cell line (A375) and potent activities towards MCF-**7** and ACHN cancer cell lines. The results of the antitumor activity of synthesized derivatives were presented in (Table 10, Chakrapani et al. [52]).

**Table 10:** Cytotoxicity data for compound 16a-j [52]

Compound	IC_50_ values, μM		
	A375	MCF-7	ACHN
16a	11.4	10.2	18.5
16b	1.22	0.23	0.11
16c	2.98	0.70	1.89
16d	14.6	19.1	6.47
16e	8.20	11.2	7.7
16f	2.70	8.41	17.6
16 g	17.7	9.7	12.2
16 h	2.20	5.98	10.6
16i	9.56	13.7	2.44
16j	0.37	1.47	0.33
Doxorubicin	5.51	2.02	0.79

**Scheme 11:**
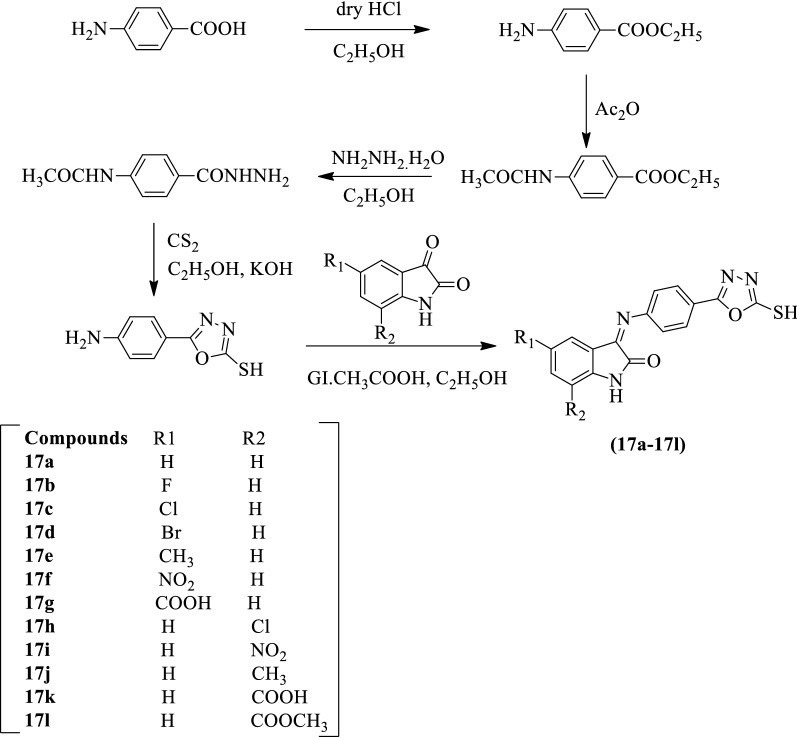
Synthesis of substituted 1,3,4-oxadiazole with p-amino benzoic acid as starting material

Gudipati et al. [53] developed *(Z)-3-[(4-(5-mercapto-1,3,4-oxadiazol-2-yl)phenyl) imino]-5 or 7-substituted indolin-2-one* (Scheme 11) and evaluated for antitumor activity by MTT assay against MCF-7, IMR-32, and HeLa tumor cell lines using cisplatin as a reference standard. The compounds **17b-17d** showed the most potent antitumor activity than the rest of other derivatives. The results of antitumor activity were summarized in (Table 11, Gudipati et al. [53]).

**Table 11:** Anticancer activity of synthesized compounds against HeLa, IMR-32 & MCF-7 cancer cells using MTT assay [53]

Compound	R_1_	R_2_	IC_50_ (μM)^*^(HeLa)	IC_50_ (μM)^*^(IMR-32)	IC_50_ (μM)^*^ (MCF-7)
Isatin			521.9	352.74	410.95
17	Intermediate		309.59	176.85	206.95
17a	H	H	25.47	30.65	33.62
17b	F	H	11.99	13.48	15.57
17c	Cl	H	12.84	15.84	16.68
17d	Br	H	10.64	12.68	16.06
17e	CH_3_	H	22.59	27.25	29.38
17f	NO_2_	H	18.60	22.51	24.48
17 g	COOH	H	17.25	20.85	22.95
17 h	H	Cl	18.69	22.51	24.92
17i	H	NO_2_	16.20	19.35	20.38
17j	H	CH_3_	15.12	18.32	20.95
17 k	H	COOH	20.36	24.28	25.98
17 l	H	COOCH_3_	19.32	23.85	25.18
Cisplatin			14.08	13.64	13.54

Values are expressed as means (n = 4)

**Scheme 12:**
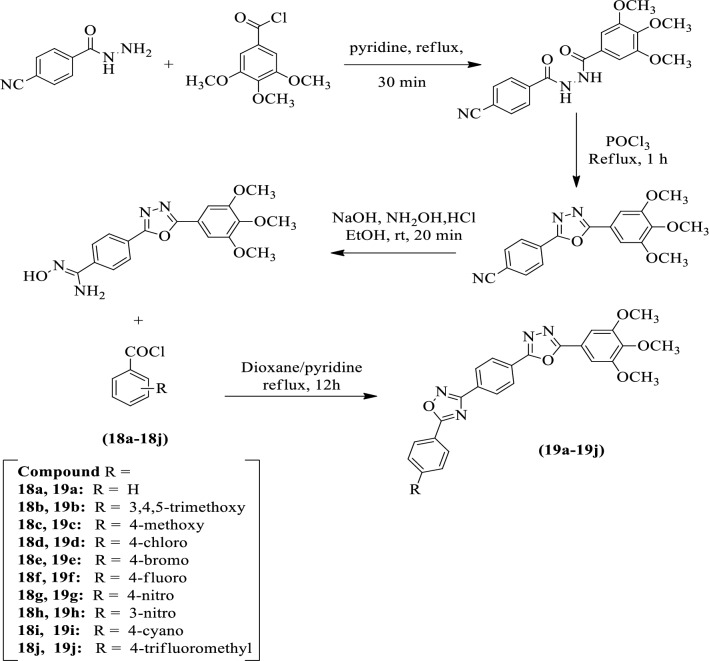
Synthesis of substituted 1,3,4-oxadiazole linked 1,2,4-oxadiazole

Polothi et al. [54] developed *5-(substituted phenyl)-3-(4-(5-(3,4,5-trimethoxyphenyl)-1,3,4-oxadiazol-2-yl)phenyl)-1,2,4-oxadiazole* by using Scheme 12 and evaluated for antitumor activity by MTT assay against MDA MB-231, MCF-7 (breast cell line), A549 **(**lung cell line) cancer cell lines using doxorubicin as a reference standard. Among the different derivatives, compounds **19b, 19g, 19h**, and **19i** showed good cytotoxic activity as compared to the reference standard. The compound **19b** with 3, 4, 5-trimethoxy group on phenyl ring shows excellent antitumor activity against human cancer cell lines such as A549 and MCF-7. The results of the antitumor activity of synthesized derivatives were showed in (Table 12, Polothi et al. [54]).

**Table 12:** In vitro cytotoxic activity [IC_50_ (μM)^a^] of compounds (19a-j) [54]

Compound	Lung cancerA549^c^	Breast cancer	
		MCF-7^b^	MDA MB-231^d^
19a	9.78 ± 0.27	34.55 ± 2.34	–
19b	0.45 ± 0.03	1.76 ± 0.34	2.11 ± 0.21
19c	3.67 ± 0.18	2.89 ± 0.67	12.76 ± 0.81
19d	4.56 ± 0.19	2.33 ± 0.56	7.34 ± 0.26
19e	13.78 ± 1.78	12.4 ± 0.79	19.5 ± 2.11
19f	34.9 ± 2.30	15.3 ± 1.72	–
19g	1.03 ± 0.17	1.23 ± 0.30	1.89 ± 0.35
19h	2.45 ± 0.23	0.34 ± 0.025	1.11 ± 0.18
19i	1.89 ± 0.38	1.90 ± 0.41	3.78 ± 0.29
19j	87.5 ± 4.67	6.30 ± 0.35	22.5 ± 1.28
Doxorubicin	2.10 ± 0.14	3.12 ± 0.17	3.41 ± 0.23

(–) not active, ^a^Each data represents as mean ± S.D values. From three different experiments performed in triplicates. MCF-7: Human breast cancer cell line. ^c^A549: Human lung cancer cell line. MDA MB-231^d^: Human breast cancer cell line

## Antitubercular activity

**Scheme 13:**
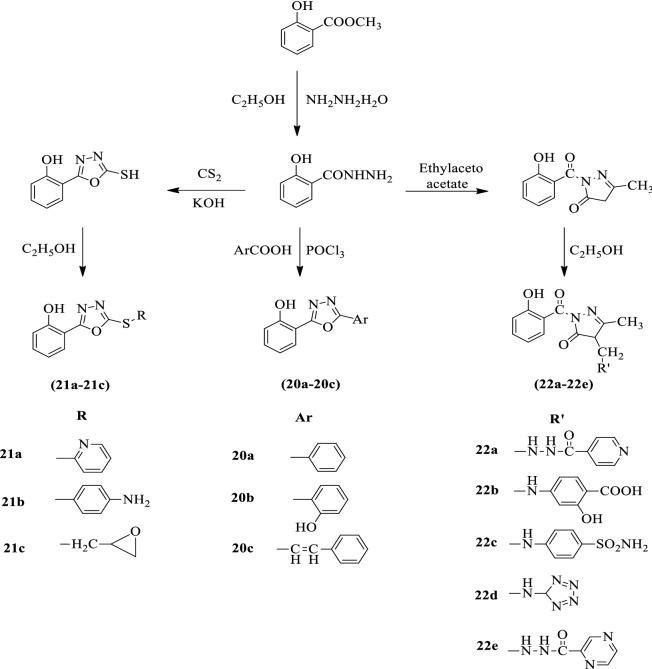
Synthesis of 1,3,4-oxadiazole derivatives

Pattan et al. [55] developed *2-(5-(substituted thio)-1,3,4-oxadiazol-2-yl) phenol* and *4-(substituted-1-ylmethyl)-1-(2-hydroxy benzoyl)-3-methyl-1H-pyrazol-5(4H)-one* by using Scheme 13. The antimycobacterial activity of the synthesized derivatives was evaluated against *Mycobacterium tuberculosis* (H_37_Rv) by MB 7H9 agar medium. Streptomycin was used as a reference standard. Compounds **20a, 21b, 22a, 22b, 22c**, and **22e** showed promising antitubercular activity. Compounds **20b, 20c**, and **22d** showed moderate activity and the results of activity were presented in (Table 13, Pattan et al. [55]).

**Table 13:** Antitubercular activity data of the synthesized compounds [55]

Compound	Antitubercular activity	
	50 μg/mL	100 μg/mL
20a	S	S
20b	R	R
20c	R	R
21a	R	R
21b	S	S
21c	R	R
22a	S	S
22b	S	S
22c	S	S
22d	R	R
22e	S	S
Streptomycin	S	S

*R* Resistant; *S* Sensitive

**Scheme 14:**
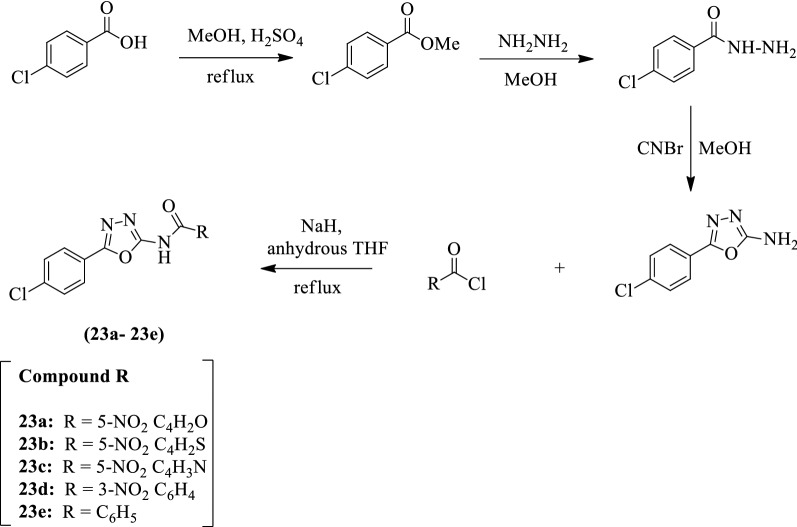
Synthesis of substituted 1,3,4-oxadiazole derivatives

Martinez et al. [44] developed *N-(5-(4-chlorophenyl)-1,3,4-oxadiazol-2-yl) substituted amide* by using Scheme 14. The antimycobacterial activity of synthesized derivatives was evaluated against different *Mycobacterium tuberculosis* strains such as 209, H37Ra, and H_37_Rv using rifampin as a reference standard. Compound **23a** shows more potent activity in comparison to the rest of other compounds. The results of the antitubercular activity of the synthesized derivatives were presented in (Table 14, Martinez et al. [44]).

**Table 14:** MIC_100_ values of 23a-e against virulent, non-virulent and RIF-resistant *M. tuberculosis* bacteria [44]

Compound	R	MIC_100_ (μg/ml) in H_37_Rv ATCC 27294	MIC_100_ (μg/ml) inH_37_Ra	MIC_100_ (μg/ml) in Mtb-209 (resistant)
23a	5-NO_2_C_4_H_2_O	7.80	1–2.00	7.8
23b	5-NO_2_C_4_H_2_S	15.60	15.60	15.60
23c	5-NO_2_C_4_H_3_O	31.25	7.8	7.8
23d	5-NO_2_C_6_H_4_	15.60	31.30	15.60
23e	5-C_6_H_5_	15.60	62.50	31.25
Rifampin	-	0.06	0.008	> 64

*M. tuberculosis* H_37_Rv ATCC 27294 reference strain; Mtb. *M. tuberculosis* H_37_Ra non-virulent strain; Mtb-209 RIF-resistant clinical isolate of *M. tuberculosis*

**Scheme 15:**
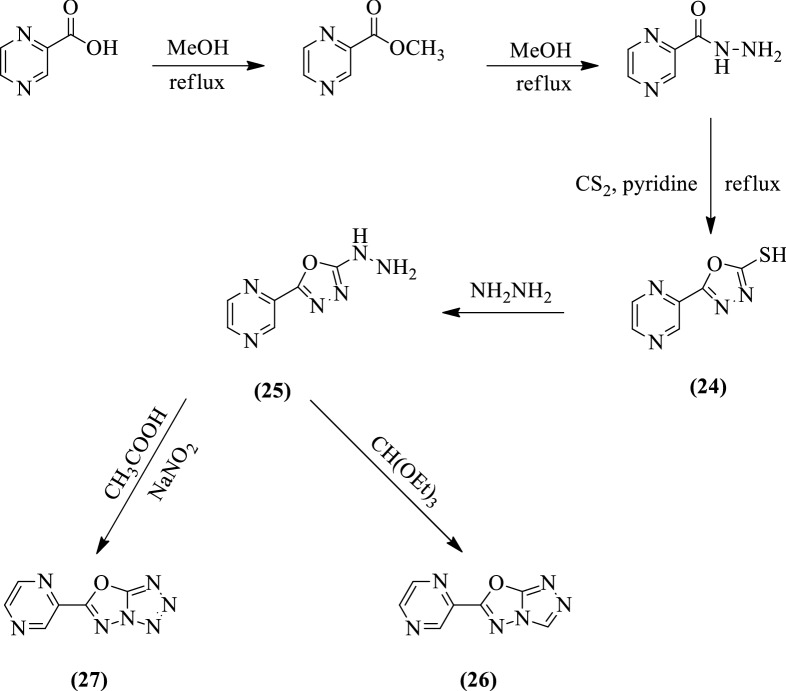
Synthesis of 1,3,4-oxadiazole linked triazole and tetrazole compounds

Das et al. [56] synthesized *6-(pyrazin-2-yl)-[1,3,4]oxadiazolo[3,2-d]tetrazole* and *6-(pyrazin-2-yl)-[1,2,4]triazolo[3,4-b][1,3,4]oxadiazole* (Scheme 15) and antimycobacterial activity of these derivatives were evaluated by (LJ) agar method against *Mycobacterium tuberculosis* H_37_Rv (MTCC200) using isoniazid and rifampicin as a reference standard. The compound **25** shows more potent antitubercular activity but still, it is lesser active than the reference standard. The results of antimycobacterial activity were showed in (Table 15, Das et al. [56]).

**Table 15:** Anti Tuberculosis activity against *Mycobacterium tuberculosis* H_37_Rv (MTCC200) [56]

Compound	MIC̽ (μg/ml)
24	> 100
25	6.25
26	50
27	50
Rifampicin	0.25
Isoniazid	0.20

*MIC* Minimum inhibitory concentration

**Scheme 16:**
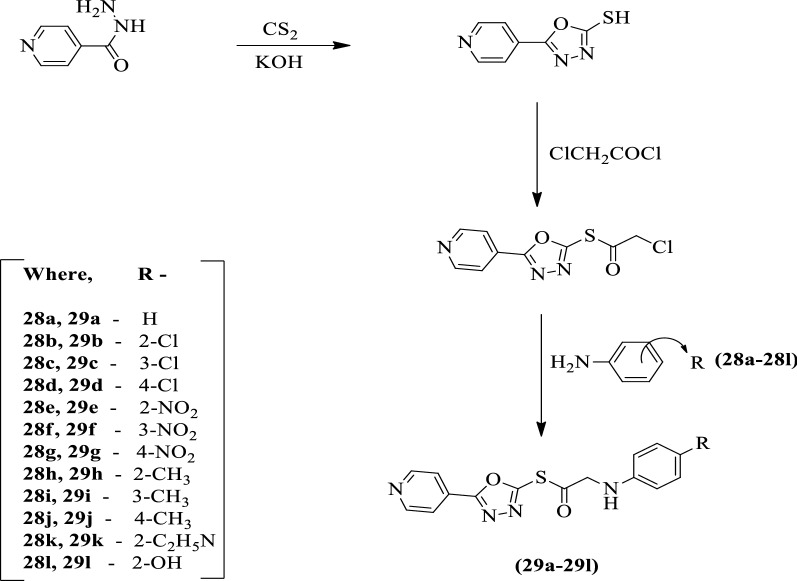
Synthesis of substituted 1,3,4-oxadiazole

Raval et al. [57] developed *S-(5-(pyridin-4-yl)-1, 3, 4-oxadiazol-2-yl)2-((substituted phenyl)amino)ethanethioate* using Scheme 16. The antitubercular activity of synthesized derivatives was evaluated against *Mycobacterium tuberculosis* H_37_Rv (ATCC27294). Rifampin was used as a reference standard. Compounds **29e, 29g**, and **29k** show better activity and exhibited > 90% inhibition. The conclusion of antimycobacterial activity was presented in (Table 16, Raval et al. [57]).

**Table 16:** Antitubercular activity of the synthesized compounds (29a-l) against *M. tuberculosis* H_37_Rv [57]

Compound	Primary screen (6.25 μg/ml)	% inhibition	Concentration (μM)	Actual MIC (μg/Ml)	Clog P̽
29a	> 6.25	64	0.0354	–	0.4996
29b	> 6.25	12	0.1640	–	1.5150
29c	> 6.25	32	0.1706	–	1.5150
29d	> 6.25	28	0.1735	–	1.5150
29e	> 6.25	92	0.0077	6.05	0.8964
29f	> 6.25	86	0.00132	5.92	0.8964
29g	> 6.25	96	0.0052	6.00	0.8964
29h	> 6.25	63	0.1130	–	0.9986
29i	6.25	62	0.1138	–	0.9986
29j	> 6.25	64	0.1133	–	0.9986
29k	> 6.25	96	0.0089	5.77	− 0.8943
29l	6.25	69	0.1184	–	− 9.1673
Isoniazid	> 6.25	98	0.025	0.05	− 0.6680

**Scheme 17:**
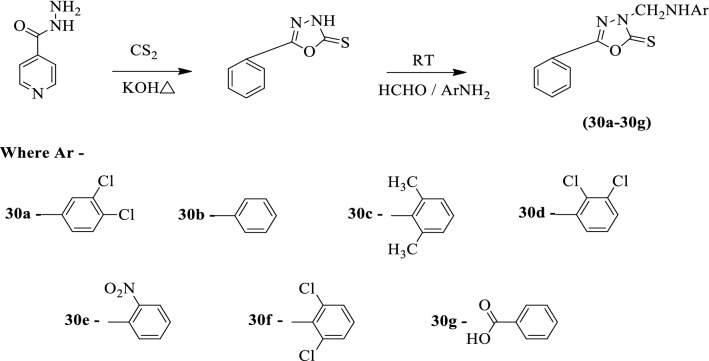
Synthesis of substituted 1,3,4-oxadiazole

Somani et al. [58] developed *3-((substituted amino) methyl)-5-phenyl-1,3,4-oxadiazole-2(3H)-thione* by using Scheme 17. The antimycobacterial activity of synthesized derivatives was evaluated against *Mycobacterium tuberculosis* H_37_Rv strain in MB 7H-9 agar medium using rifampicin as a reference standard. The conclusion of the antimycobacterial activity of synthesized derivatives was presented in (Table 17, Somani et al. [58]).

**Table 17:** Antitubercular activity of the synthesized compounds (30a-3g) against *M. tuberculosis* H_37_Rv [58]

Compound	Antitubercular activity		
	25 (µg/ml)	50 (µg/ml)	100 (µg/ml)
30a	R	R	S
30b	R	S	S
30c	S	S	S
30d	S	S	S
30e	S	S	S
30f	R	R	S
30g	R	R	S
Rifampicin	S	S	S

**Scheme 18:**
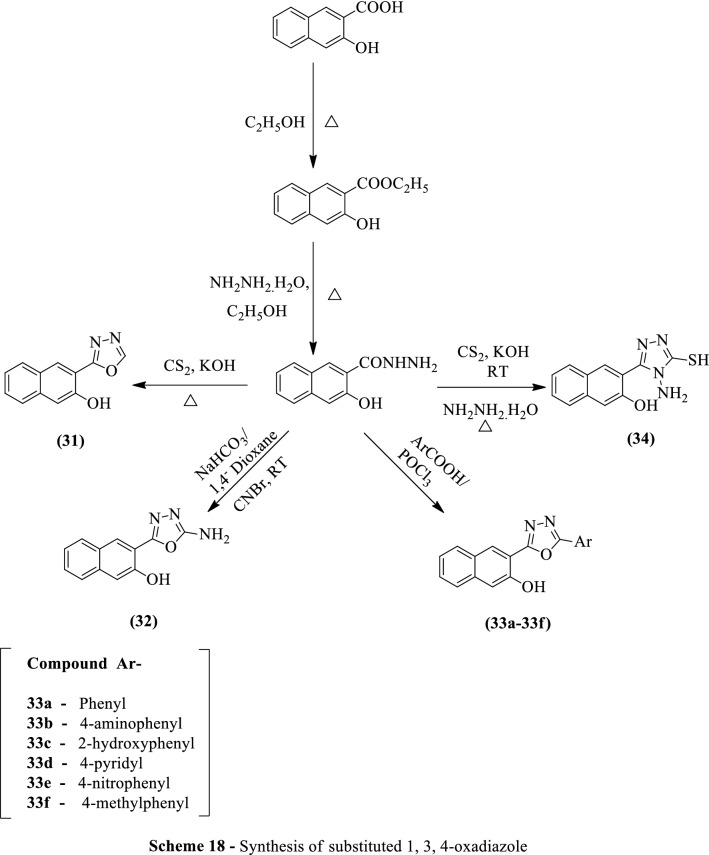
Synthesis of substituted 1,3,4-oxadiazole

Gavarkar et al. [59] developed *3-(5-substituted-1,3,4-oxadiazol-2-yl) naphthalen-2-ol* using Scheme 18. These derivatives were evaluated for antimycobacterial activity by tube dilution method against *Mycobacterium tuberculosis* H_37_Rv strain using MB 7H-9 agar broth. Streptomycin and Pyrazinamide were used as a reference standard. Compounds **31, 33c**, and **33d** exhibited good antitubercular activity as compare to reference standards and the results were summarized in (Table 18, Gavarkar et al. [59]).

**Table 18:** Antitubercular activity of the titled compounds against *M. tuberculosis* H_37_Rv [59]

Compound	Antitubercular activity		
	5 (µg/mL)	10 (µg/mL)	25 (µg/mL)
31	R	S	S
32	R	R	R
33a	R	R	R
33b	R	R	R
33c	R	S	S
33d	S	S	S
33e	R	R	R
33f	R	R	R
34	R	S	R
Streptomycin	R	S	S
Pyrazinamide	R	S	S

## Antiviral activity

**Scheme 19:**
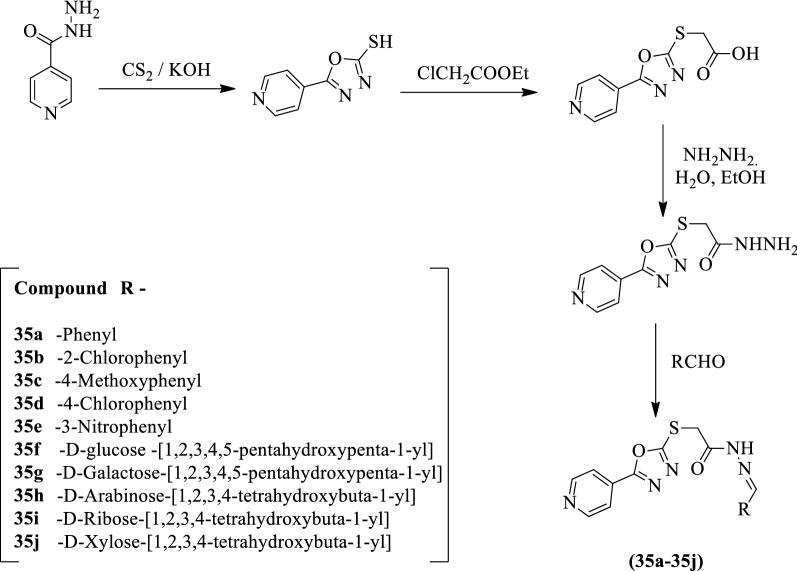
Synthesis of substituted 1,3,4-oxadiazole

Somani et al. [47] developed *N'-substituted-2-((5-(pyridin-4-yl)-1,3,4- oxadiazol-2-yl)thio)acetohydrazide* (Scheme 19) and evaluated for antiviral activity against a different type of strains such as HIV-2 ROD and HIV-1 IIIB using MTT assay in MT-4 cells. Nevirapine was used as a reference standard. These derivatives were also evaluated for cytotoxic activity using MTT assay in uninfected MT-4 cells. The results of synthesized derivatives were expressed as CC_50_, IC_50_, and SI values which were summarized in Table 19a. The results of the antiviral activity of synthesized derivatives against other viruses in (HEL) and (Vero) culture were reported in (Table 19b, c, Somani et al. [47]).

**Table 19:** (a) Anti HIV activity of synthesized compounds. (b) Cytotoxicity and antiviral activity of titled compounds in Vero cell cultures. (c) Cytotoxicity and antiviral activity of titled compounds in HEL cell cultures [47]

Panel (a)						
Compound	HIV I (μg/ml)		SI	HIV II (μg/ml))		SI
	IC50	CC50		IC50	CC50	
35a	> 50	= 50	< 1	> 57	= 57	< 1
35b	> 65	= 65	< 1	> 60	= 60	< 1
35c	> 125	> 125	X1	> 125	> 125	X1
35f	> 125	> 125	X1	> 38	> 125	> 3
35 g	> 125	> 125	X1	> 125	> 125	X1
35 h	> 125	> 125	X1	> 125	> 125	X1
35i	> 125	> 125	X1	> 125	> 125	X1
35j	> 125	> 125	X1	> 125	> 125	X1
Nevirapine(μM)	> 0.25	> 200	> 800	–	–	–
DDI (μM)	> 5.37	> 529	> 98	2.71	> 529	> 195

Panel (b)						
Compound	Minimum cytotocic concentration^a^ (μg/mL)	EC_50_^b^ (μg/mL)				
		Para-influenza-3 virus	Retrovirus	Sindbis virus	Coxasacide B4 virus	Punta Toro virus
35a	20	> 20	> 20	> 20	> 20	> 20
35b	100	> 20	> 20	> 20	> 20	> 20
35c	100	> 20	> 20	> 20	> 20	> 20
35f	> 100	> 100	> 100	> 100	> 100	> 100
35 g	> 100	> 100	> 100	> 100	> 100	> 100
35 h	> 100	> 100	> 100	> 100	> 100	> 100
35i	> 100	> 100	> 100	> 100	> 100	> 100
35j	> 100	> 100	> 100	> 100	> 100	> 100
Ribavirin (μM)	> 250	146	250	> 250	> 250	146

Panel (c)					
Compound	Minimum cytotocic concentration^a^ (μg/mL)	EC_50_^b^ (μg/mL)			
		Herpes simplex virus-1	Herpes simplex virus-2	Vaccinia virus	Vesicular stomatitis virus
35a	> 100	50	100	45	> 100
35b	100	> 20	> 20	> 20	> 20
35c	> 100	> 100	> 100	> 100	> 100
35f	> 100	> 100	> 100	> 100	> 100
35g	> 100	> 100	> 100	> 100	> 100
35h	> 100	> 100	> 100	> 100	> 100
35i	> 100	> 100	> 100	> 100	> 100
35j	> 100	> 100	> 100	> 100	> 100
Brivudin (μM)	> 250	0.04	50	2	250
Cidofovir (μM)	> 250	1	1	2	> 250
Ganciclovir (μM)	> 100	0.02	0.07	> 100	> 100

^a^Concentration required to cause a microscopically detectable alteration of normal cell morphology, ^b^Concentration required to reduce virus-induced cytopathogenicity by 50%

**Scheme 20:**
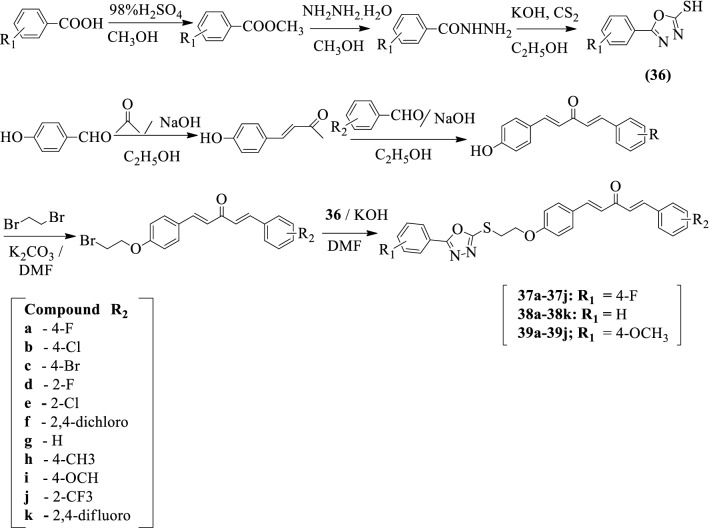
Synthesis of substituted 1,3,4-oxadiazole with benzoic acid as starting material

Gan et al. [25] developed *(1E, 4E)-1-(substituted)-5-(4-(2-((5-substituted)-1,3,4-oxadiazol-2-yl)thio)ethoxy)phenyl)Penta-1,4-dien-3-one* by using Scheme 20. The antiviral activity of synthesized compounds was evaluated against (TMV) using ribavirin as a reference standard. Among the synthesized derivatives, compounds **37a, 37c, 37f, 38a, 38b, 38c, 38d, 38e, 38f, 38g, 38h, 38i, 39e**, and **39f** exhibited potent curative activities as compared to a reference standard. Compounds **37a-37h** and **38a-38g** showed good protective activity against TMV as compared to the reference standard. Moreover, compounds **37a**-**37g, 38c, 38e, 38f, 38g, 38i**, and **39a-39j** showed better activities as compared to the positive control. Among them, compound **38f** shows the best curative, inactivation, and protective activity as compare to the reference standard. The results of the antiviral activity of different derivatives were showed in (Table 20, Gan et al. [25]).

**Table 20:** Antiviral activity of the titled compounds [25]

Compound	R_1_	R_2_	Curative activity(%)	Protective activity(%)	Inactivation activity(%)
37a	4-F	4-F	43.2 ± 2.1	55.9 ± 1.7	84.4 ± 1.2
37b	4-F	4-Cl	25.9 ± 1.8	52.5 ± 1.5	88.4 ± 0.8
37c	4-F	4-Br	45.6 ± 1.9	67.9 ± 3.9	74.8 ± 1.3
37d	4-F	2-F	31.1 ± 2.3	68.4 ± 3.2	83.4 ± 1.6
37e	4-F	2-Cl	23.7 ± 3.1	56.8 ± 2.6	56.2 ± 1.9
37f	4-F	2,4-Di-Cl	52.9 ± 4.5	65.1 ± 3.2	83.5 ± 2.7
37g	4-F	H	28.2 ± 1.1	52.9 ± 0.7	74.5 ± 0.9
37h	4-F	4-CH_3_	19.2 ± 0.9	60.5 ± 1.1	61.3 ± 0.8
37i	4-F	4-OCH_3_	27.5 ± 2.1	50.0 ± 1.5	61.4 ± 1.0
37j	4-F	2-CF_3_	28.3 ± 2.3	47.5 ± 2.4	60.2 ± 1.7
38a	H	4-F	45.8 ± 1.8	61.5 ± 2.9	69.1 ± 1.2
38b	H	4-Cl	44.1 ± 2.5	55.7 ± 1.6	59.4 ± 2.5
38c	H	4-Br	47.2 ± 3.6	53.8 ± 3.9	83.1 ± 2.4
38d	H	2-F	38.1 ± 2.6	66.3 ± 1.9	70.1 ± 2.0
38e	H	2-Cl	41.1 ± 4.2	61.5 ± 3.1	75.6 ± 2.1
38f	H	2,4-Di-Cl	49.8 ± 3.9	69.2 ± 2.1	90.4 ± 2.8
38g	H	H	20.9 ± 2.1	66.7 ± 2.8	78.0 ± 2.5
38h	H	4-CH_3_	48.1 ± 3.6	57.5 ± 2.7	72.7 ± 3.3
38i	H	4-OCH_3_	40.6 ± 3.2	58.4 ± 3.8	79.3 ± 4.1
38j	H	2-CF_3_	35.5 ± 1.7	50.5 ± 1.9	56.8 ± 2.1
39a	4-OCH_3_	4-F	20.8 ± 1.2	44.0 ± 0.9	83.0 ± 1.1
39b	4- OCH_3_	4-Cl	18.4 ± 0.9	34.4 ± 1.1	87.1 ± 1.8
39c	4- OCH_3_	4-Br	34.8 ± 2.1	41.1 ± 3.6	82.3 ± 5.1
39d	4- OCH_3_	2-F	25.4 ± 1.7	35.8 ± 1.4	81.3 ± 2.1
39e	4- OCH_3_	2-Cl	43.5 ± 2.2	46.1 ± 2.6	77.7 ± 2.0
39f	4- OCH_3_	2,4-Di-Cl	43.9 ± 2.4	49.6 ± 1.8	85.6 ± 1.9
39g	4- OCH_3_	H	37.8 ± 1.6	42.5 ± 2.0	78.8 ± 2.1
39h	4- OCH_3_	4-CH_3_	26.5 ± 1.2	42.1 ± 2.1	86.3 ± 5.4
39i	4- OCH_3_	4-OCH_3_	35.1 ± 1.5	41.5 ± 1.8	81.5 ± 2.6
39j	4- OCH_3_	2-CF_3_	30.5 ± 2.1	49.3 ± 2.3	77.9 ± 4.5
38k	H	2,4-Di-F	55.4 ± 2.8	71.3 ± 1.9	85.2 ± 4.0
Ribavirin			37.9 ± 1.9	51.8 ± 2.3	72.9 ± 2.4

**Scheme 21:**
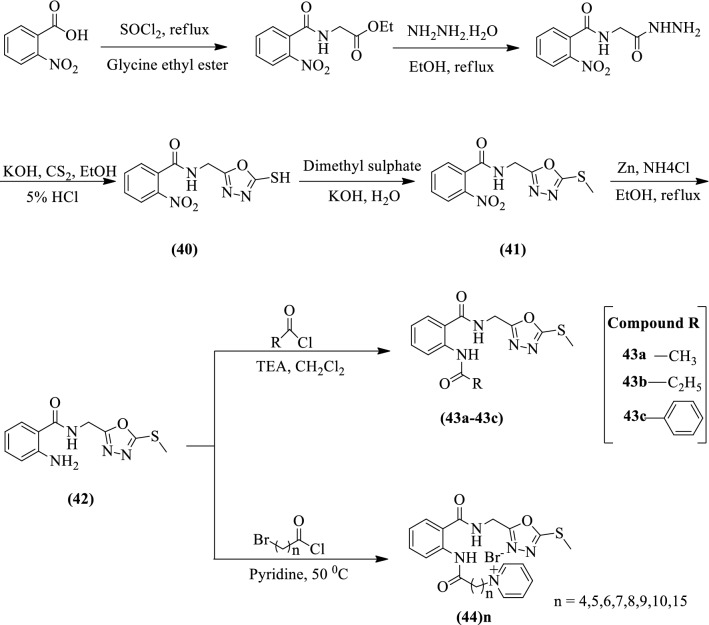
Synthesis of 1,3,4-oxadiazole derivatives with 2-nitrobenzoic acid as starting material

Wang et al. [1] developed *N-((5-mercapto-1,3,4-oxadiazol-2-yl)methyl)-2-nitro benzamide, N-((5-(methylthio)-1,3,4-oxadiazol-2-yl)methyl)-2-nitro benzamide, 2-amino-N-((5-(methylthio)-1,3,4-oxadiazol-2-yl)methyl)benzamide* and *2-(substituted)-N-((5-(methylthio)-1,3,4-oxadiazol-2-yl) methyl)benzamide* (Scheme 21) and evaluated for antiviral activity. NNM was used as a reference standard. Among the synthesized derivatives, compounds **44**_**6**_**, 44**_**8**_, and **44**_**15**_ showed a more potent activity than the reference standard. The position of the substituent’s also affected the antiviral activity and the results of antiviral activity were represented in (Table 21, Wang et al. [1]).

**Table 21:** Anti-TMV activities of titled compounds at 500 μg/mL in vivo [1]

Compounds	Rate (%)		Compounds	Rate (%)	
	Curative activity	Protective activity		Curative activity	Protective activity
40	38.5 ± 1.2	35.2 ± 3.1	44_8_	60.0 ± 5.6	36.4 ± 1.0
41	36.9 ± 5.1	14.4 ± 2.9	44_9_	26.9 ± 2.9	43.3 ± 3.0
42	26.8 ± 5.2	54.5 ± 2.9	44_10_	48.7 ± 5.1	25.2 ± 2.9
43a	22.3 ± 6.4	54.6 ± 5.2	44_15_	51.9 ± 3.0	45.6 ± 4.2
43b	47.2 ± 2.8	38.8 ± 4.5	40’	41.8 ± 1.0	41.7 ± 1.7
43c	44.8 ± 9.5	36.8 ± 0.8	41’	17.5 ± 1.2	32.2 ± 1.6
44_4_	7.1 ± 1.7	51.2 ± 7.6	42’	17.7 ± 1.2	42.6 ± 2.2
44_5_	37.4 ± 3.5	27.8 ± 5.5	43_2_’	49.3 ± 2.0	19.6 ± 2.4
44_6_	50.6 ± 4.7	42.9 ± 2.5	44_10_’	33.9 ± 1.3	20.2 ± 1.0
44_7_	37.1 ± 3.3	23.5 ± 1.1	44_15_’	35.3 ± 2.3	19.3 ± 0.8
NNM	54.2 ± 2.9	65.7 ± 2.2			

**Scheme 22:**
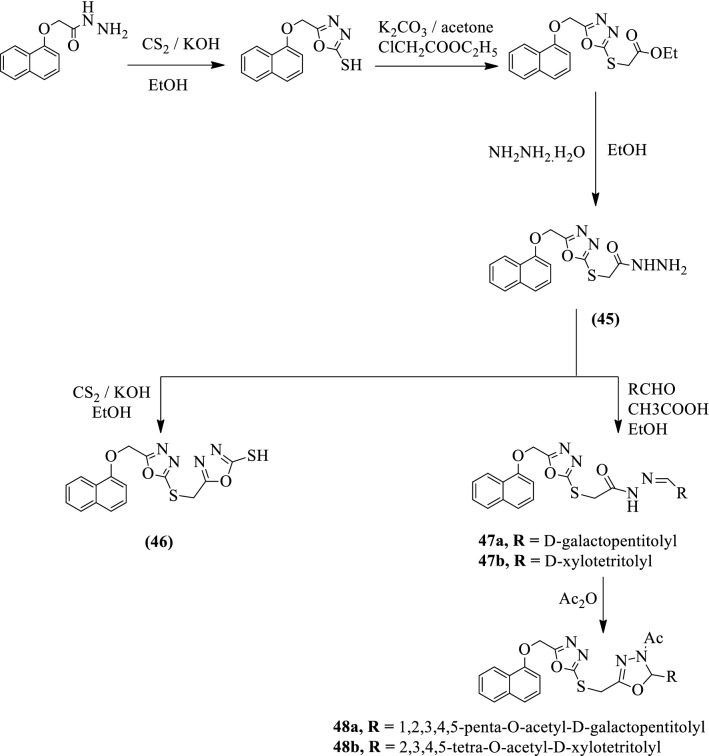

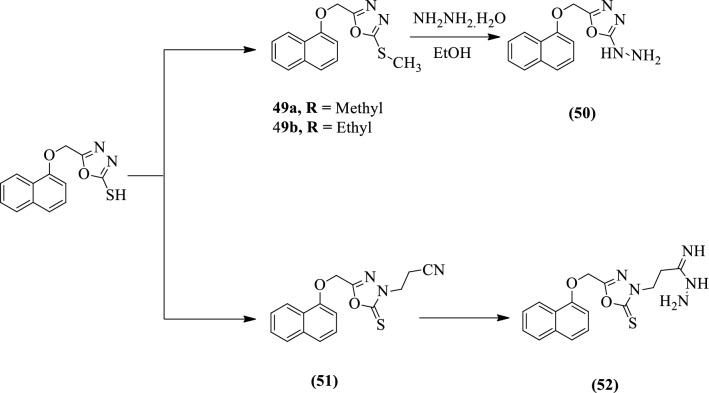
**a** Synthesis of disubstituted 1,3,4-oxadiazoles.**b** Synthesis of hydrazide and imidrazone of 1,3,4-oxadiazoles

EI-Sayed et al. [60] developed *1,2,3,4,5-Penta-O-acetyl-D-galactopentitolyl* and *2,3,4,5-tetra-O-acetyl-D-xylotetritolyl, hydrazide,* and *imidrazone of 1,3,4-oxadiazole* by using Scheme 22a, b respectively. The antiviral activity of synthesized derivatives was evaluated as reverse transcriptase inhibitors with fresh human peripheral blood mononuclear cells. Compound **47b** shows good antiviral activity followed by compounds **45** and **49a.** Compounds **48b** and **52** showed moderate activity while **47a** and **48a** showed the weakest activity among the series of tested compounds. The results of the antiviral activity of synthesized derivatives were presented in (Table 22, EI-Sayed et al. [60]).

**Table 22:** HIV inhibition activities (reverse transcriptase inhibitor) with therapeutic index [60]

Compound	EC50 (μM)	IC50 (μM)	Therapeutic index
45	3.24. 10^–3^	1.88	2.88. 10^–7^
47a	1.1. 10^–5^	12.89	66.24. 10^–8^
47b	5.26. 10^–4^	1.44	3.15. 10^–7^
48a	5.23. 10^–4^	12.44	5.78. 10^–6^
48b	1.56. 10^–3^	3.11	3.45. 10^–6^
49a	3.81. 10^–3^	2.12	8.14. 10^–6^
52	2.72. 10^–3^	2.9	5.12. 10^–6^

## Antioxidant activity

**Scheme 23:**
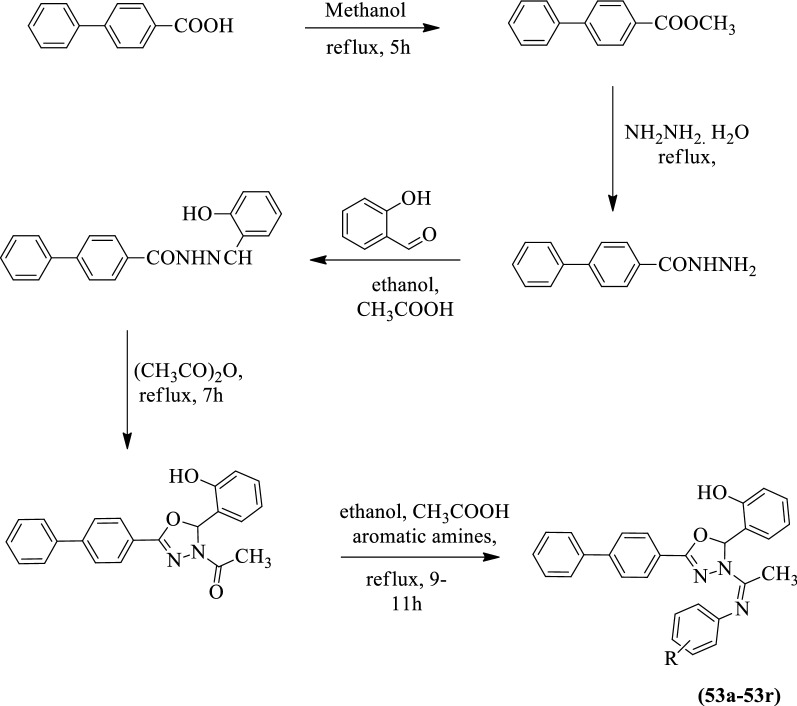
Synthesis of substituted 1,3,4-oxadiazole with 4-biphenyl carboxylic acid as starting material

Malhotra et al. [46] developed *(Z)-2-(5-[(1, 1-biphenyl)-4-yl]-3-(1-((substituted)imino) ethyl)-2,3-dihydro-1,3,4-oxadiazol-2yl)phenol* (Scheme 23) and evaluated for antioxidant activity in terms of hydrogen peroxide scavenging activity. The results of the antioxidant activity of the synthesized derivatives were presented in (Table 23, Malhotra et al. [46]).

**Table 23:** Hydrogen peroxide scavenging activity of synthesized compounds [46]

Compound	Scavenging of hydrogen peroxide at different concentration (%)		
	100 (µg/ml)	300 (µg/ml)	500 (µg/ml)
53a	41.55	39.84	41.22
53b	46.34	44.55	45.77
53c	51.11	48.12	44.59
53d	41.92	42.33	41.72
53e	45.65	46.19	45.91
53f	51.21	43.12	39.57
53g	39.58	42.61	43.18
53h	43.45	41.37	45.27
53i	41.88	45.19	48.11
53j	47.52	54.15	53.18
53k	45.35	50.27	52.15
53l	51.15	52.27	58.18
53m	45.87	41.37	41.93
53n	42.98	39.72	39.57
53o	41.03	43.06	44.14
53p	51.62	52.18	52.91
53q	54.18	53.76	57.36
53r	49.87	51.35	48.74
BHA	63.27	66.19	68.25
Ascorbic acid	51.47	53.45	55.38

**Scheme 24:**
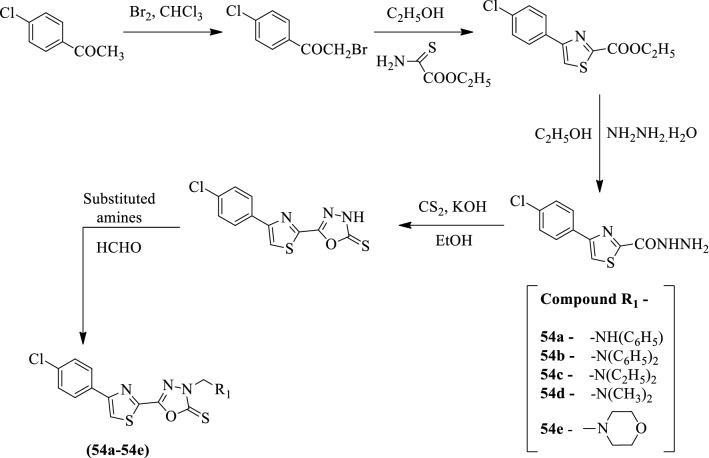
Synthesis of substituted 1,3,4-oxadiazole

Rahul R. et al. [8] synthesized *5-(4-(4-chlorophenyl)thiazol-2-yl)-3-(substituted benzyl) -1,3,4-oxadiazole-2(3H)-thione* by using Scheme 24 and evaluated for antioxidant activity by different methods such as Hydrogen peroxide scavenging, Nitric oxide scavenging, and DPPH assay. In DPPH assay compound **54c** shows more significant activity in comparison to ascorbic acid. In other methods such as hydrogen peroxide and nitric oxide scavenging assay, compound **54c** gives more potent activity than the rest of the other compounds but was not significant as compare to the results obtained in the DPPH assay. This shows that compound **54c** gives more potent antioxidant activity as compared to the rest of the synthesized compounds. The results of the antioxidant activity of synthesized derivatives were presented in (Table 24, Rahul R. et al. [8]).

**Table 24:** (a) DPPH assay of synthesized compounds. (b) Nitric oxide scavenging of synthesized compounds. (c) Hydrogen peroxide scavenging of synthesized compounds

Compound	% Scavenging activity at different concentrations					IC_50_
	20 (µg/ml)	40 (µg/ml)	60 (µg/ml)	80 (µg/ml)	100 (µg/ml)	
Panel (a)						
54a	39.94 ± 0.521	59.14 ± 0.652	61.38 ± 0.631	63.59 ± 0.245	65.34 ± 0.534	29.7
54b	46.63 ± 0.342	49.7 ± 0.352	57.51 ± 0.421	60.51 ± 0.634	62.65 ± 0.453	43.3
54c	44.86 ± 0.245	62.22 ± 0.214	64.66 ± 0.341	65.82 ± 0.372	67.76 ± 0.215	26.7
54d	44.64 ± 0.234	53.89 ± 0.123	62.73 ± 0.223	64.02 ± 0.321	66.92 ± 0.431	27.1
54e	47.34 ± 0.235	48.16 ± 0.516	49.54 ± 0.461	52.98 ± 0.371	55.75 ± 0.297	61.3
Ascorbic acid	49.38 ± 0.515	67.03 ± 0.541	75.78 ± 0.223	91.92 ± 0.561	95.34 ± 0.111	21.3
Panel (b)						
54a	34.83 ± 0.527	40.63 ± 0.654	43.87 ± 0.691	52.15 ± 0.215	53.11 ± 0.514	72.1
54b	27.34 ± 0.372	29.81 ± 0.352	38.25 ± 0.421	42.55 ± 0.639	50.54 ± 0.450	98.3
54c	33.57 ± 0.243	44.97 ± 0.211	48.69 ± 0.348	52.35 ± 0.442	53.15 ± 0.218	66.2
54d	33.28 ± 0.232	44.40 ± 0.128	45.70 ± 0.224	52.01 ± 0.331	54.29 ± 0.481	69.8
54e	26.67 ± 0.295	29.30 ± 0.506	44.95 ± 0.411	51.98 ± 0.381	52.07 ± 0.297	70.6
Ascorbic acid	47.53 ± 0.624	63.44 ± 0.521	84.28 ± 0.623	90.53 ± 0.411	93.56 ± 0.221	25.2
Panel (c)						
54a	35.75 ± 0.612	44.97 ± 0.237	55.19 ± 0.226	65.93 ± 0.662	67.14 ± 0.653	47.1
54b	34.01 ± 0.563	43.51 ± 0.464	58.83 ± 0.152	60.48 ± 0.353	62.50 ± 0.452	49.1
54c	34.24 ± 0.263	46.06 ± 0.533	58.82 ± 0.623	62.12 ± 0.621	63.63 ± 0.236	43.3
54d	33.93 ± 0.235	46.81 ± 0.516	56.52 ± 0.532	59.89 ± 0.623	61.39 ± 0.425	45.6
54e	34.48 ± 0.342	44.88 ± 0.345	55.57 ± 0.173	56.61 ± 0.535	58.63 ± 0.654	50.6
Ascorbic acid	44.53 ± 0.526	64.65 ± 0.653	71.74 ± 0.36	89.22 ± 0.621	96.19 ± 0.456	26.9

IC_50_ values in µg/ml for samples were determined using ED50 plus V 1.0 software. Data are the mean of three or more experiments and reported as mean ± standard error of the mean (SEM)

**Scheme 25:**
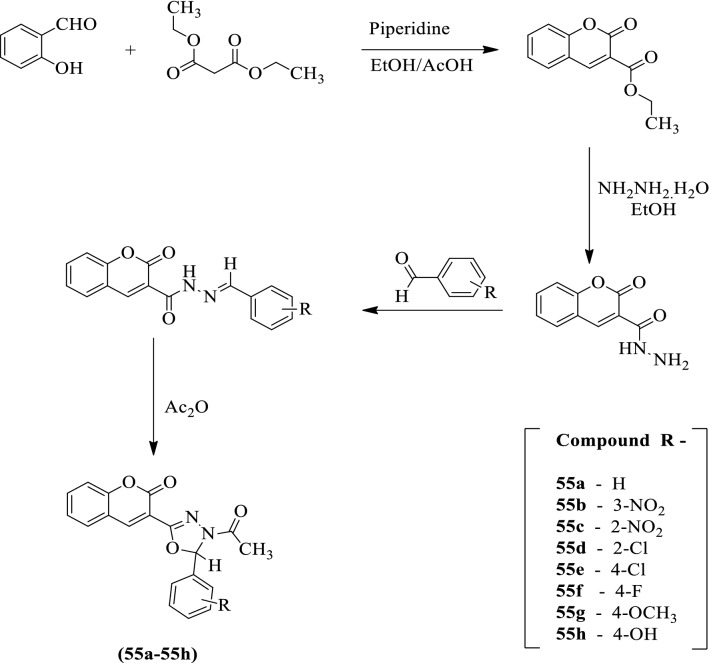
Synthesis of substituted 1,3,4-oxadiazole with 2-hydroxy benzaldehyde carboxylic acid as starting material

Dureja [61] developed *3-(4-acetyl-5-(substituted phenyl)-4, 5-dihydro-1,3,4-oxadiazol-2-yl)-2H-chromen-2-one* (Scheme 25) and evaluated for antioxidant activity by using DPPH assay. Ascorbic acid was used as a reference standard and the results were summarized in (Table 25, Dureja [61]).

**Table 25:** Antioxidant activity of synthesized compounds by DPPH method [61]

Compound	% Scavenging activity	IC_50_
55a	19.97–85.95	47.47 ± 2.473
55b	3.07–64.92	197.96 ± 2.454
55c	7.4–48.75	> 500
55d	13.87–77.45	60.93 ± 1.560
55e	12.60–85.95	> 500
55f	14.70–69.70	130.8 ± 3.602
55g	4.9–74.77	90.26 ± 2.442
55h	6.85–69.42	91.70 ± 2.778
Ascorbic acid	44.95–95.5	12.7 ± 0.68

## Conclusion

In this present review article, we have summarized different pharmacological activities of 1,3,4-oxadiazole containing compounds. From this study, we have found that 1,3,4-oxadiazole containing compounds can be synthesized by various kinds of synthetic routes, and these derivatives having a wide range of biological activities such as antitumor, antitubercular, antimicrobial, antiviral and antioxidant, etc. This review article established the fact that 1,3,4-oxadiazole as useful templates for further modification or derivatization to design more potent biologically active compounds.

## Data Availability

All data are provided in the manuscript or cited in the references.

## Abbreviations

CNS: Central Nervous System
FDA: Food and Drug Administration
MTT: 3-(4, 5-Dimethylthiazol-2-yl)-2,5-diphenyltetrazolium bromide
IC_50_: Half maximal inhibitory concentration
L.J: Lowenstein-Jensen
MB: Middle brook
HeLa: Henrietta Lacks
MIC: Minimum inhibitory concentration
HIV: Human immunodeficiency virus
CC_50_: Half maximal cytotoxic concentration
SI: Selectivity index
HEL: Human embryonic lung fibroblast
VERO: Verda reno (means green kidney)
TMV: Tobacco mosaic virus
DPPH: 2, 2-Diphenyl-1-picrylhydrazyl
MTCC: Microbial type cell cultures
